# Phenotypic covariation predicts diversification in an adaptive radiation of pupfishes

**DOI:** 10.1002/ece3.11642

**Published:** 2024-08-07

**Authors:** Julia C. Dunker, Michelle E. St. John, Christopher H. Martin

**Affiliations:** ^1^ Department of Integrative Biology University of California Berkeley California USA; ^2^ Museum of Vertebrate Zoology University of California Berkeley California USA; ^3^ Present address: Department of Biology University of Oklahoma Norman Oklahoma USA

**Keywords:** adaptive radiation, craniofacial traits, flexibility, P matrix, phenotypic variation and covariation

## Abstract

Phenotypic covariation among suites of traits may constrain or promote diversification both within and between species, yet few studies have empirically tested this relationship. In this study, we investigate whether phenotypic covariation of craniofacial traits is associated with diversification in an adaptive radiation of pupfishes found only on San Salvador Island, Bahamas (SSI). The radiation includes generalist, durophagous, and lepidophagous species. We compared phenotypic variation and covariation (i.e., the P matrix) between (1) allopatric populations of generalist pupfish from neighboring islands and estuaries in the Caribbean, (2) SSI pupfish allopatric lake populations with only generalist pupfish, and (3) SSI lake populations containing the full radiation in sympatry. Additionally, we examine patterns observed in the P matrices of two independent lab‐reared F2 hybrid crosses of the two most morphologically distinct members of the radiation to make inferences about the underlying mechanisms contributing to the variation in craniofacial traits in SSI pupfishes. We found that the P matrix of SSI allopatric generalist populations exhibited higher levels of mean trait correlation, constraints, and integration with simultaneously lower levels of flexibility compared to allopatric generalist populations on other Caribbean islands and sympatric populations of all three species on SSI. We also document that while many craniofacial traits appear to result from additive genetic effects, variation in key traits such as head depth, maxilla length, and lower jaw length may be produced via non‐additive genetic mechanisms. Ultimately, this study suggests that differences in phenotypic covariation significantly contribute to producing and maintaining organismal diversity.

## Graphical abstract

This study explores the relationship between diversification and trait covariation using an endemic adaptive radiation of pupfishes. We find evidence suggesting that strong covariation between traits may constrain pupfish diversification, and that nonadditive genetic variation may be responsible for variation in important craniofacial traits in the system.

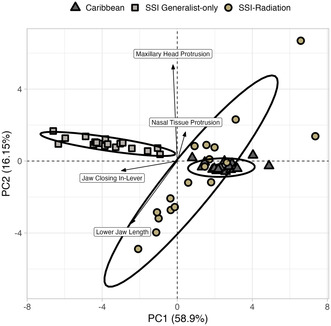

## 1 INTRODUCTION

Understanding the factors that produce and maintain diversity is a fundamental goal of evolutionary biology. Genetic and environmental variation interact to produce observed phenotypic variation, and many studies have successfully identified the specific genes, alleles, or selective pressures that have resulted in divergence between closely related groups (Colosimo et al., 2005; Endler, 1995; Gao et al., 2009; Gomi et al., 2007; Martin et al., 2019; Occhialini et al., 2012; Steingrímsson et al., 2006; Torres‐Dowdall et al., 2012; Winchell et al., 2016; Zhan et al., 2010). Yet, many organisms, such as *Howea* palms, Andean *Coeligena* hummingbirds, *Amphilophus* and Barombi Mbo cichlids, and *Vidua* indigobirds, display high degrees of phenotypic variation and diversification but exhibit limited genetic variation or live in similar environments where selective pressures are assumed to be the same (Barluenga et al., 2006; Martin et al., 2015; Palacios et al., 2019; Papadopulos et al., 2011; Richards et al., 2018; Savolainen et al., 2013). The presence of phenotypic variation and diversification across several taxa, in the absence of obvious genetic or environmental divergence, suggests that the relationship between diversity and additional axes of variation should be explored.

The observed relationships between traits (hereafter referred to as phenotypic variation and covariation) are an additional complex phenotype that can provide valuable insight into diversification. Phenotypic variance and covariance are typically quantified as a matrix (hereafter referred to as the P matrix), which is an established method for determining the multivariate phenotypes present within a population (Cheverud et al., 1989; Lande & Arnold, 1983). Estimates and comparisons of P matrices have been used to make inferences about various measures of organismal performance (e.g., feeding or locomotion) and are particularly useful for quantifying levels of integration, identifying modules, and understanding how populations will respond to selective pressures (Goswami & Polly, 2010; Haber, 2015; Kane & Higham, 2015; Pavlicev et al., 2009; Reichert & Höbel, 2018). Although it was previously assumed that P matrices were constant across time (Lande, 1979), more recent work – including empirical studies – now supports that (1) P matrices are subject to change and (2) evolutionary forces can act on variation at the level of the P matrix to produce convergent or divergent phenotypes (Blankers et al., 2015; Evans et al., 2017; Kolbe et al., 2011; Michaud et al., 2020; Roff & Mousseau, 2005; Steppan et al., 2002; Selz et al., 2014). Therefore, investigating the relationship between traits as an additional axis of diversity may provide new avenues for uncovering the genetic and environmental variation that produces it.

Investigating the relationship between variation in the P matrix and diversification can also provide insights into the underlying mechanisms contributing to phenotypic variation. The P matrix has been used as a proxy for the genetic variance–covariance matrix (i.e., the G matrix) for some time and is generally more easily attainable than direct estimates of covariation between genes (Cheverud, 1988; Roff, 1995). Comparing P matrices between groups can provide insight into if and how the genetic architecture or shared developmental pathways of traits responds to different selective pressures (Cheverud, 1995; Kolbe et al., 2011; Marroig & Cheverud, 2001; Schluter, 1996). Furthermore, investigating the P matrix of hybrid offspring of divergent groups can provide more specific information regarding the genetic basis of traits of interest because the laws of independent assortment, segregation, and assumptions of additivity provide a null expectation for how phenotypic variation should shift across generations (Falconer, 1996; Roff, 1997). For instance, deviations from additive expectations in F1 hybrids and backcrosses were used as evidence that non‐additive genetic variation was likely a large contributor to beak shape divergence in species of *Geospiza* finches. Deviations from null expectations observed in hybrids have also been used to infer X‐linkage of acoustic traits in field crickets (Blankers et al., 2015) and to infer the contributions of gene–gene and gene–environment interactions in diverging populations of flour beetles (Drury et al., 2013).

In summary, comparing P matrices, within and between species or groups, can provide additional information on how we may expect populations to respond to selective pressures. This information, in turn, can help us make further inferences about the diversification process and can provide us with clues as to the genetic architecture, developmental pathways, or mechanisms that are responsible for variation in traits. To gain these additional insights, we must empirically investigate (1) if differences in variance and covariance between phenotypic traits are associated with diversification and (2) if P matrix attributes of hybrids deviate from the null expectations of the laws of independent assortment, segregation, and assumptions of additivity.

The *Cyprinodon* pupfish system is excellent for investigating whether phenotypic covariation is associated with diversity for two reasons: first, the pupfish system contains at least three species that display extensive phenotypic divergence. *Cyprinodon variegatus* (hereafter referred to as the generalist pupfish) has an extremely large range that stretches along the Atlantic coast from North America, throughout the Caribbean, and into northern portions of South America (Echelle & Echelle, 2020; Hildebrand, 1917). In the interior hypersaline lakes of the San Salvador Islands, Bahamas (hereafter referred to as SSI), however, there is a radiation of pupfishes that not only includes the widespread generalist pupfish species but also additional snail‐eating (*C. brontotheroides*) and scale‐eating (*C. desquamator*) specialist species. Previous work has documented the behavioral, morphological, and physiological diversity that characterizes each of these species, but their most obvious axes of divergence are in their craniofacial musculoskeletal elements (Heras & Martin, 2022; Martin, 2016; Martin et al., 2017; Martin & Wainwright, 2013a, 2013b, 2013c; Palominos et al., 2023; St. John, Dixon, & Martin, 2020; St. John, Holzman, & Martin, 2020). Briefly, generalist pupfish jaws are similar to those of other Cyprinodontiformes; snail‐eating pupfish exhibit an expanded dorsal head of the maxilla, and scale‐eating pupfish have a significantly larger oral jaw apparatus (Hernandez et al., 2018; Martin & Wainwright, 2011).

Second, despite the observed phenotypic diversity, patterns of Caribbean‐wide genetic diversity and environmental variation do not appear to sufficiently explain the pattern of trophic specialist species restricted to a single island. Although specialist pupfish are endemic to the hypersaline lakes of SSI, generalist pupfish populations are found across the Atlantic coasts of the Americas, in lakes across other Caribbean islands, and even in allopatry on SSI (Martin, 2016; Martin & Wainwright, 2011). Furthermore, the hypersaline lake environments on SSI are so far not detectably different than other islands in biotic diversity, dietary composition of pupfish populations, geologic composition, or lake areas (Martin, 2016), suggesting that environmental differences alone are not solely responsible for pupfish diversification. Similarly, phylogenetic evidence suggests that radiating and non‐radiating populations of pupfish on SSI likely share a common ancestor that resembles the Caribbean generalist pupfish (Martin & Feinstein, 2014; Martin, 2016). While there is potentially adaptive genetic differentiation between generalist, snail‐eating, and scale‐eating pupfishes, a recent study found similar levels of genetic diversity between radiating and non‐radiating lineages of pupfish and that nearly all the adaptive genetic variation found in specialists exists everywhere as standing genetic variation (Patton et al., 2022; Richards & Martin, 2017, 2022; Richards et al., 2021). The incredible trait diversification rates of the pupfish radiation on SSI, paired with our current understanding of the available genetic and environmental variation, indicate that this system is a good candidate for investigating the relationship between phenotypic covariation and diversification.

In this study, we (1) determined if radiating lineages on SSI display unique multivariate phenotypes and covariation between traits, which may have promoted their diversification relative to neighboring island generalist populations, and (2) compared multivariate phenotypes and covariation among F2 hybrid offspring to make inferences about the underlying mechanisms of craniofacial traits in pupfishes. We calculated and compared variance–covariance matrices for 18 craniofacial traits for (1) allopatric populations of generalists from neighboring islands and estuaries across the Caribbean, (2) SSI allopatric generalist populations, and (3) sympatric lake populations of all three species found on SSI. We further calculated variance–covariance matrices for F2 hybrid offspring of scale‐eating and snail‐eating crosses from two radiating populations of pupfish on SSI to address our second question (St. John, 2024). We predicted that sympatric populations containing the full radiation of pupfishes on SSI would differ in their multivariate phenotypes and covariation structure among traits relative to allopatric generalist populations. We also predicted that the multivariate phenotypes and covariation between traits observed in F2 hybrids would not deviate from assumptions of additivity among traits.

## 2 METHODS

### 2.1 Focal populations

**FIGURE 1:**
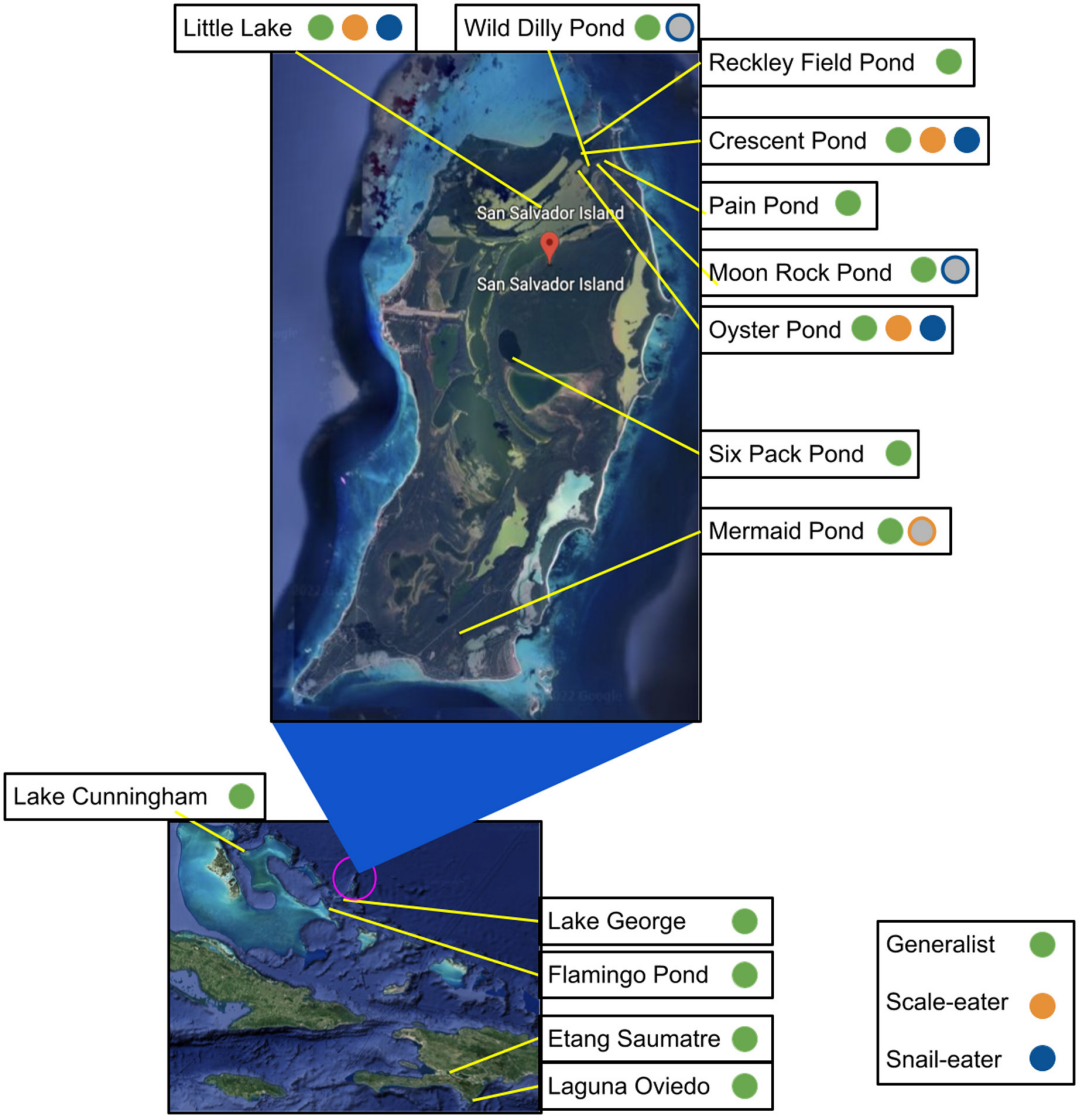
Map highlighting the location of study populations and species. The inset designated by the blue triangle provides a large view of San Salvador Island, Bahamas. Circles filled with color indicate species sampled from each location (green: generalist pupfish, orange: scale‐eating pupfish, blue: snail‐eating pupfish). Gray circles outlined in color represent species that may be present in each population but were not sampled in this study. Map images from Google Earth (2023).

The goal of this study was to compare the P matrices of allopatric generalist pupfish populations to sympatric populations of pupfish, containing both generalist and specialist species, on SSI and to estimate the P matrices of F2 hybrid pupfish to make inferences about the underlying mechanisms of craniofacial traits on SSI. To that end, we measured and compared craniofacial traits of fish from: five Caribbean populations of pupfish that contained only generalist species (Lake Cunningham (New Providence Island, Bahamas), Flamingo Pond (Acklins, Bahamas), Lake George (Rum Cay Island, Bahamas), Etang Saumatre (Dominican Republic), and Laguna Oviedo (Dominican Republic)); six SSI populations that did not contain all three species of pupfishes within the SSI radiation, and instead contained only generalist or one additional specialist species (Wild Dilly Pond, Reckley Field Pond, Pain Pond, Moon Rock Pond, Six Pack Pond, and Mermaid Pond); and three SSI populations of pupfish that contained all three species of pupfishes in sympatry (Crescent Pond, Little Lake, and Oyster Pond), although only generalist and scale‐eater specimens were available for measurement from Oyster Pond (Table 1; Figure 1). A portion of the specimens from the above ponds were initially collected, measured, and analyzed in a previously published article (Martin, 2016), and complete collection details can be found there. Additional justification and information about the use and categorization of these populations can be found in Appendix A. For simplicity, we collectively refer to the five Caribbean populations of pupfish as the “Caribbean” group/population (*N* = 61), the six populations that do not contain the full pupfish radiation as the “generalist‐only” group/population (*N* = 85), and the three populations containing all three pupfish species in sympatry as the “radiating” group/population (*N* = 42).

**TABLE 1:** Sample sizes of measured individuals across Caribbean populations, San Salvador Island generalist‐only populations, and San Salvador Island radiating populations.

Population	Location	Generalist	Snail‐eater	Scale‐eater	F2 hybrids
Caribbean					
Etang Saumatre	Dominican Republic	16			
Flamingo Pond	Acklins, Bahamas	7			
Lake Cunningham	New Providence Island, Bahamas	10			
Lake George	Rum Cay, Bahamas	15			
Laguna Oviedo	Dominican Republic	13			
Total: 61					
SSI Generalist‐only					
Mermaid Pond	San Salvador Island, Bahamas	16			
Moon Rock Pond	San Salvador Island, Bahamas	21			
Pain Pond	San Salvador Island, Bahamas	14			
Reckley Field Pond	San Salvador Island, Bahamas	11			
Six Pack Pond	San Salvador Island, Bahamas	15			
Wild Dilly Pond	San Salvador Island, Bahamas	8			
Total: 85					
SSI Radiation					
Crescent Pond	San Salvador Island, Bahamas	4	3	5	301
Little Lake	San Salvador Island, Bahamas	4	8	7	194
Oyster Pond	San Salvador Island, Bahamas	3		8	
Total: 42					

*Note*: Overall, we measured craniofacial traits for 188 wild‐caught individuals across the Caribbean (including San Salvador Island and 496 lab‐reared F2 hybrids). We measured 61 individuals from five populations (i.e., “Caribbean”), 85 individuals from six populations on San Salvador Island that do not contain the full radiation of pupfish species (i.e., “SSI generalist‐only”), and 42 individuals from three of the radiating populations on San Salvador Island (i.e., “SSI radiation”).

To make inferences about the potential mechanisms underlying craniofacial traits on SSI, we measured traits of F2 scale‐eater X snail‐eater hybrid offspring and estimated phenotypic variation and covariation of these traits. We produced F2 hybrids by first crossing a single male snail‐eater with a single female scale‐eater to produce F1 offspring. We repeated this process independently for the Crescent Pond and Little Lake populations. At least four F1 offspring from each population were then allowed to interbreed to produce F2 hybrids. In total, we produced and measured 301 F2 hybrids from Crescent Pond and 194 hybrids from Little Lake (Table 1). These measurements were used for a separate QTL mapping study of these crosses (St. John, 2024).

When comparing F2 hybrid phenotypes and P matrices, we assume a simple additive model of inheritance (Falconer, 1996; Roff, 1997). The assumptions of this model include: (1) that there are two alleles per locus; (2) that Mendelian laws of segregation are adhered to; (3) that loci across the genome are in linkage equilibrium; and (4) that there are only additive genetic effects (i.e., no dominance or epistatic effects). With these assumptions, we expect that the distribution of phenotypic traits in the F2 generation should follow a 1:2:1 ratio, where intermediate phenotypes are most common and phenotypes aligning with either parental phenotype are less common and to be uniformly distributed, and we expect covariation between traits to correspond to recombination events, which, for the sake of simplicity, we assume to be uniformly distributed across the genome. To investigate deviations from these assumptions, we estimate expectations of additivity by calculating the average of parental traits from the F0 generation and expectations of variation within traits using the parental trait values and hybrid population sample sizes (Tables E1, E2, E3 in Appendix E).

### 2.2 Clearing and staining

Clearing and staining specimens promotes observation of the specimens' skeletal structures and specifically allows us to measure internal skeletal traits. We therefore cleared and stained 683 fish specimens in preparation for future measurements. We fixed pupfish specimens in 95% ethanol, skinned them, and then immersed the specimens in 5% buffered formalin and double‐stained the specimens with alizarin red and alcian blue as outlined in Dingerkus & Uhler (1977). After, we suspended the specimens in glycerin and took lateral and dorsal photos with a Canon EOS 60D digital SLR for downstream digital measurements. The camera was mounted to a stand and positioned approximately 6–8 inches above specimens. Each photo included a grid of known length and/or ruler to ensure that measurements could be scaled correctly in future analyses.

### 2.3 Collection of morphological data

**FIGURE 2:**
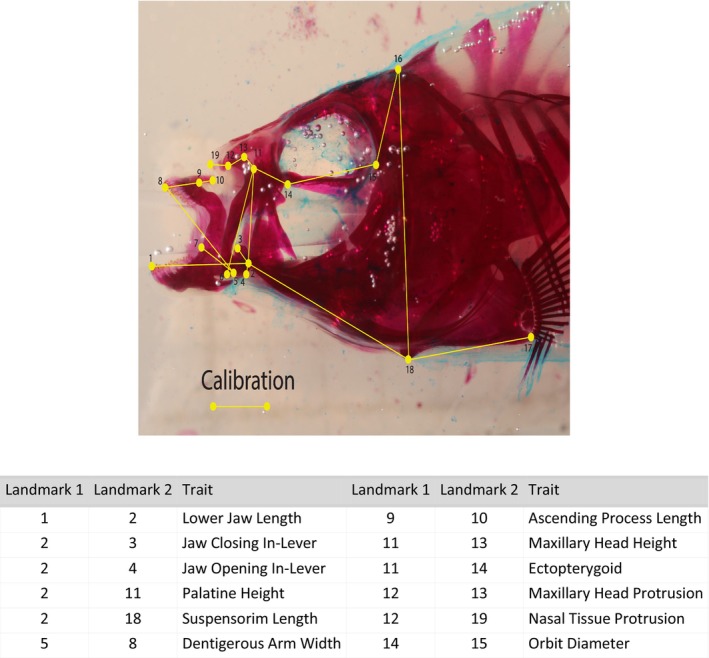
Landmark location and craniofacial trait names displayed on the lateral view of a cleared and stained pupfish specimen. Landmarks from Martin (2016).

We measured morphological traits to compare the phenotypes and P matrices of pupfishes across species and populations. We measured 18 craniofacial traits detailed in Martin et al. (2017) (Figure 2). We specifically focused on craniofacial traits for this study because jaw diversity is the primary axis of diversification in this system and is hypothesized to be adaptive for each specialist's unique ecological niche (Hernandez et al., 2018; Martin et al., 2017). We measured traits using the program DLTdv8a (Hedrick, 2008), which outputs X and Y coordinates for each landmark and subsequently calculates linear distances from these coordinates. We standardized each cranial trait measurement by performing a linear model with each trait measurement as the response variable and log‐transformed standard length as the predictor variable using the lme4 package in R 4.1.1 (Bates et al., 2014; R Core Team, 2021). We extracted the residuals for each individual and used these values in all further analyses.

### 2.4 The effect of sample size on estimates of covariation

While the sample sizes presented in this study are modest, they are within the ranges suggested as sufficient in the literature. For example, Cheverud's analysis (1988) suggests that 40 individuals is a large enough sample size for estimating genetic variance from phenotypic variance. More recent simulation‐based studies suggest that adequate sample sizes likely vary depending on the statistics being used and the properties of one's dataset, but show that sample sizes of 16–32 are large enough to accurately estimate most common statistics associated with variance–covariance datasets (Grabowski & Porto, 2017; Watanabe, 2022). There are also several previously published empirical studies examining the properties of variation‐covariation with sample sizes similar to our own (Blankers et al., 2015; Goswami & Polly, 2010; Polly, 2005). To further ensure that our sample size was adequate for detecting differences in means between groups, we performed power analyses to calculate the minimum sample size required across a range of correlations and effect sizes with 80% power. We also performed Monte Carlo simulations across a range of hypothetical trait values and standard deviations to determine at which sample size estimates of variation stabilized. Full methodologies and results can be found in Appendix B, but overall, we found that our sample sizes were sufficient for detecting differences between groups even at small effect sizes (*η* ^2^ > 0.02) and that estimates of trait variation stabilized at 20 individuals.

### 2.5 Investigating differences in multivariate phenotypes between groups and hybrid populations

We used a Bayesian mixed‐effects model to investigate if trait means varied between Caribbean, SSI generalist‐only, and SSI radiating groups while accounting for the effects of species and population. We used the brms function from the BRMS package to fit a model with a student's *T* distribution and uninformative priors (Bürkner, 2017). The model included the 18 trait measurements as the multivariate response variable, group type and species designation as fixed effects, and population ID as a random effect.

We fit a Bayesian fixed‐effects model with a student's *T* distribution and uninformative priors to investigate differences in trait values between Crescent Pond and Little Lake F2 hybrids. This model included the 18 trait measurements as the multivariate response variable and the population as a fixed effect. While testing for differences in trait values between hybrid groups is not a primary goal of this study, detected differences could indicate population‐level differences in the genetic architecture of these traits. Furthermore, the output of this model provides estimates of uncertainty around average trait values which were used to investigate deviations from null expectations. Specifically, we used these estimates to determine which phenotypes deviated from additive trait value expectations (Table E4 in Appendix E).

For both models, we extracted posterior samples and estimated the median values and 95% highest density intervals (HDI) to make inferences about differences in trait values between the levels of our fixed effects using functions from the brms and emmeans packages (Lenth, 2023).

### 2.6 Construction of P matrices (variance–covariance matrices) and statistical analyses

We used the CalculateMatrix function from the EvolQG 0.2‐9 package in R (Melo et al., 2016) to construct a variance–covariance P matrix for the following groups: (1) SSI generalist‐only; (2) SSI radiation; (3) Caribbean; (4) Crescent Pond F2 hybrids; and (5) Little Lake F2 hybrids. These matrices describe the variance both within a trait and the covariation between traits for a given group and are the primary unit of comparison in the following analyses. We did not use any phylogenetic correction because within‐lake populations of each species are sometimes more closely related than species across lakes (Martin & Feinstein, 2014). To visualize similarities and differences between P matrices of (1) Caribbean, SSI generalist‐only, and SSI radiating pupfish groups and (2) F2 hybrids from Crescent Pond and Little Lake, we performed two separate principal component analyses. These PC analyses were covariance‐based and included the variance and covariance estimates for the 18 craniofacial traits (prcomp function; R Core Team, 2021; Figures 4 and 8). For each of the PC analyses, we also calculated the correlation between loadings and PC axes 1 and 2 to determine (1) which loadings were most closely aligned with the variation along a given axis and (2) which loadings were most similar to one another. We also calculated the contributions of each group (i.e., Caribbean, generalist‐only, radiation, Crescent Pond F2s, or Little Lake F2s) and trait toward the patterns observed in the analyses using the get_pca_var and get_pca_ind functions from the factoextra package (Kassambara & Mundt, 2020).

We used the MeanMatrixStatistics function to estimate: autonomy, constraints, flexibility, mean squared correlation, and respondability for each matrix, and the PCAsimilarity function to estimate similarity between matrices (EvolQG 0.2‐9; Melo et al., 2016). We also used the integration.Vrel and compare.ZVrel functions (geomorph 4.0.6; Adams et al., 2023; Baken et al., 2021) to quantify and compare eigenvalue variance between matrices as an estimate of morphological integration (i.e., relative eigenvalue variance or Vrel; Conaway & Adams, 2022; Pavlicev et al., 2009). A detailed description of each measurement can be found in Table 2. Most of the above functions provide point estimates to describe attributes of a single matrix or attributes of a comparison of matrices; however, point estimates do not allow for statistical inferences. We therefore used bootstrap resampling (iterations = 100) to estimate 95% confidence intervals around point estimates and to make direct comparisons between the P matrices of each focal group. The exception to this is the results from comparing V*rel*, which natively performs a two‐sample z‐test to determine significance.

**TABLE 2:** Descriptions of the different statistics used to compare the P matrices of focal groups.

Statistic	Description
Autonomy	Proportion of variance that is aligned with a selection gradient and is separate from variation in other directions
Constraints	Mean correlation between the response vector to random selection variants and the matrix's first principal component, which largely constrains the phenotype due to its accounting for much of a species' phenotypic variation
Flexibility	Measure of how closely the response of a species aligns with the different random selection gradients
Mean squared correlation	Average of the correlation coefficients, corresponding to the overall association between the traits measured
Respondability	Pace of the change in population mean when a population is under directional selection
PCA similarity	Measure of similarity of the matrices' variation by incorporating the relative similarity of the matrices' corresponding principal components
Integration	Measure of the strength of morphological integration quantified using the relative Eigen index

*Note*: These statistics come from the EvolQG package (Melo et al., 2016) and the geomorph package (Adams et al., 2023; Baken et al., 2021), and complete details can be found there. Briefly, the autonomy, constraint, flexibility, mean squared correlation, respondability, and integration statistics provide point estimates that serve as descriptions of a single group's P matrix. On the other hand, PCA similarity statistics provide estimates of similarity between two P matrices.

Finally, to make inferences about the genetic architecture and relationship between craniofacial traits, we used the variance and covariance values from the P matrices of Crescent Pond and Little Lake F2 hybrids to compare: (1) estimates of variance for each population; (2) covariation across traits within and between populations; (3) regression coefficients across traits within and between populations; and (4) squared correlation coefficients across traits within and between populations. We used a paired *t*‐test to investigate differences in variance between populations and used linear models with either covariation, regression coefficients, or squared correlation coefficients as the response variable and population, trait, and their interaction as fixed effects. We used AIC scores to compare linear models including and excluding the interaction term and moved forward with the best‐fitting model.

## 3 RESULTS

### 3.1 SSI radiating populations have larger craniofacial trait values than Caribbean or SSI‐generalist‐only populations

**FIGURE 3:**
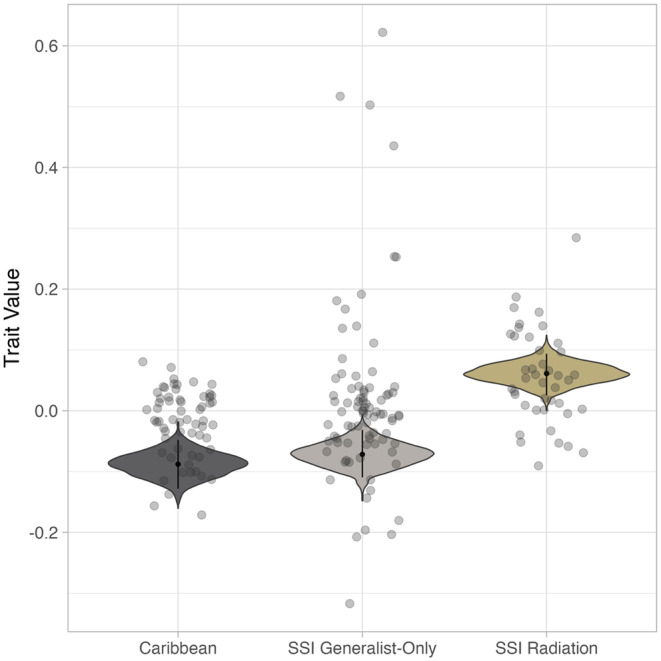
The multivariate trait values of SSI radiating pupfish are on average larger than the multivariate trait values of SSI generalist‐only and Caribbean pupfish groups. Violin plots display the posterior draws from a Bayesian mixed effects model with the 18 craniofacial trait values as the response variable, pupfish group and species designations as fixed effects, and population ID as a random effect. Black points and lines represent the median and 95% HDI for each group. Dots show average trait values for individuals.

The Bayesian mixed‐effects model results suggested that, on average, the SSI radiating group had larger trait values (mean trait value: 0.06, 95% HDI: 0.03, 0.09) than either the Caribbean (mean trait value: −0.09, 95% HDI: −0.13, −0.05) or SSI generalist‐only groups (mean trait value: −0.07, 95% HDI: −0.11, −0.03; Figure 3). Model estimates for each trait indicated that this pattern was reflected in 11 of the 18 traits. Two traits, ectopterygoid and lower jaw size, deviated slightly from this pattern. The SSI radiating group had larger estimates of ectopterygoid size compared to the Caribbean group but did not significantly differ from the SSI generalist‐only group. On the other hand, lower jaw sizes significantly varied between all three groups, with the SSI radiating group having larger lower jaw sizes than the SSI generalist‐only group, which in turn had larger lower jaw sizes compared to the Caribbean group. The remaining five of the 18 traits showed no significant differences between groups (Table D1 in Appendix D).

**FIGURE D1:**
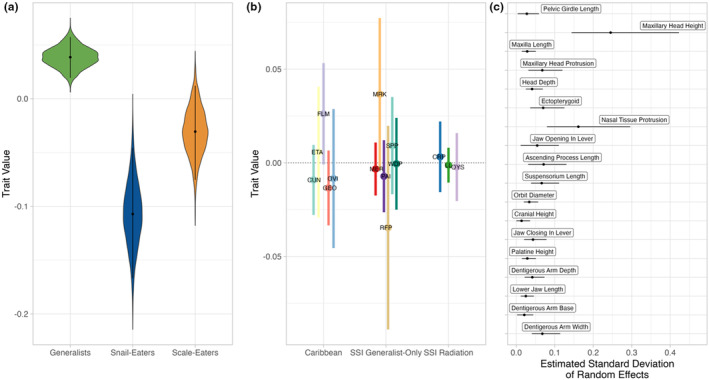
Results of the Bayesian mixed model displaying (a) differences in average trait value for generalists, snail‐eaters, and scale‐eaters, (b) average trait value and standard deviations for each population, and (c) the estimated standard deviation due to the random effect of population on each of the traits. In panel a, violin plots display the posterior draws from a Bayesian mixed effects model with the 18 craniofacial trait values as the response variable, pupfish group and species designations as fixed effects, and population ID as a random effect. Black points and lines represent the median and 95% HDI for each species. For panel b, points represent the mean value for each population, while the colored lines show the 95% confidence interval around these estimates. For panel c, points show the average standard deviation estimate for each trait, while the black lines show the 95% confidence intervals around these estimates.

We had an a priori expectation that some trait sizes would vary between species, and we therefore included species designation as a fixed effect in the model to account for this. Overall, we found that average trait values varied between all three species such that generalist values (mean trait value: 0.039, 95% HDI: 0.020, 0.058) were greater than scale‐eater values (mean trait value: −0.031, 95% HDI: −0.075, 0.013), which in turn were greater than snail‐eater values (mean trait value: −0.11, 95% HDI: −0.17, −0.046; Figure D1a in Appendix D). This hierarchical pattern, however, was not present when investigating specific traits, suggesting that the increased average trait value associated with generalists is a statistical artifact of the multivariate analysis. A more complex pattern is indeed revealed when we examine the variation between species for individual traits (Table D2 in Appendix D). Generalists had significantly greater estimates of cranial height, orbit diameter, head depth, and pelvic girdle length compared to both specialists, but also exhibited greater maxillary head height estimates compared to scale‐eaters and greater ascending process lengths compared to snail‐eaters. On the other hand, snail‐eaters showed a significant reduction in all three measurements of the dentigerous arm, palatine height, jaw closing in‐lever, and maxilla length compared to both generalists and scale‐eaters. Finally, only two traits showed a simultaneous shift in both specialist estimates: lower jaw length and maxillary head protrusion. Scale‐eaters had the highest estimates of lower jaw length, followed by generalists, while snail‐eaters had the smallest estimates. This pattern was reversed for maxillary head protrusion size, as snail‐eaters had the largest estimates (Table D2 in Appendix D).

Finally, we investigated the effects of population in our model and found that populations did not have significantly different trait value estimates (Figure D1b in Appendix D). We also examined the variability of estimates due to population ID for each craniofacial trait. The standard deviation reported for 16 of the 18 traits was below 0.1, indicating that populations generally have similar trait values. The exceptions were maxillary head height and nasal tissue protrusion, which had higher standard deviations of 0.25 and 0.16, respectively (Figure D1c in Appendix D).

### 3.2 SSI generalist‐only populations exhibit unique P matrix properties that are not observed in Caribbean or SSI radiating populations

**FIGURE 4:**
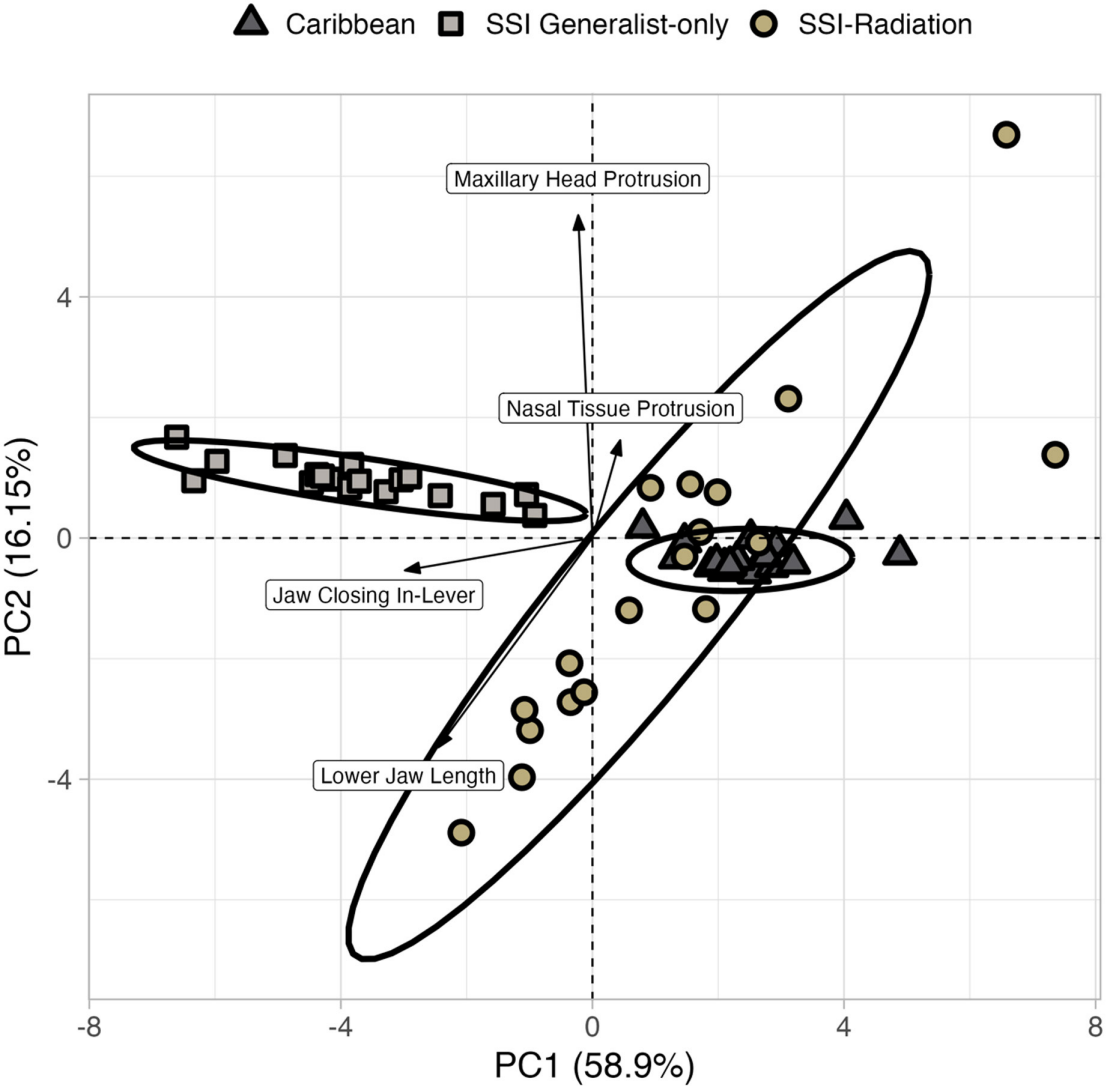
Results of a principal components analysis calculated with the variance and covariance estimates for 18 craniofacial traits for the Caribbean, SSI generalist‐only, and SSI radiating populations. PC1 and PC2 explain 59% and 16% of the variance, respectively. Shapes and colors indicate the three focal groups (dark gray triangle: Caribbean group, light gray square: SSI generalist‐only group, gold circle: SSI radiating group), and ellipses represent 95% confidence intervals per group. Arrows display four craniofacial traits that are most strongly correlated with PC1 (jaw closing in‐lever and lower jaw length) and PC2 (maxillary head protrusion and nasal tissue protrusion). San Salvador Island generalist‐only group clusters separately from the Caribbean and San Salvador Island radiating groups along PC1.

**FIGURE 5:**
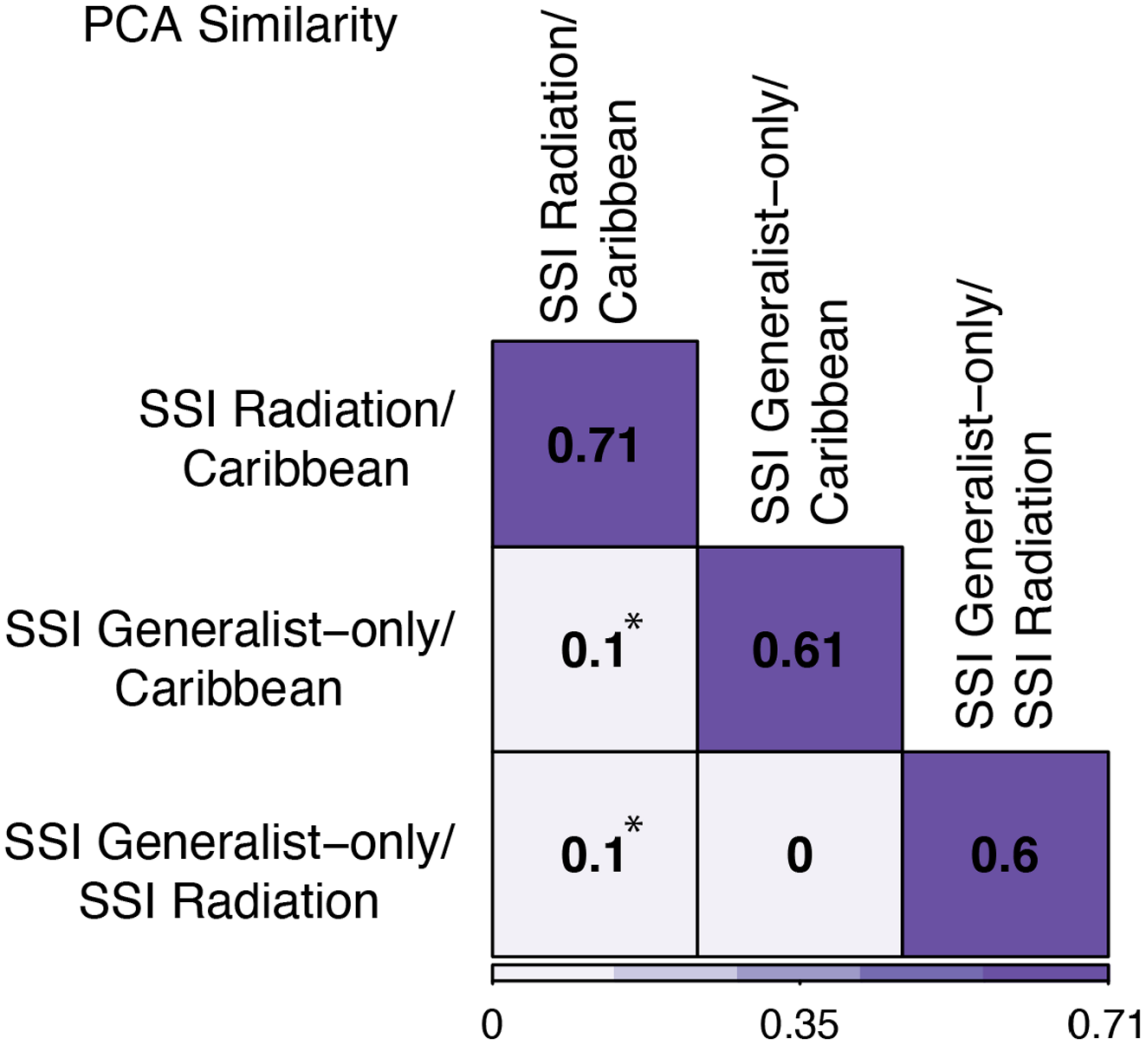
Caribbean pupfish populations and SSI radiating populations have the highest estimates of similarity, while SSI generalist‐only populations have the lowest estimates of similarity. Results of matrix comparisons using the PCA similarity method. The squares along the longest diagonal show the mean values of the P matrix metrics for the corresponding comparisons. The remaining squares show the differences between the two comparisons. Fill colors represent low (light purple) and high (dark purple) values within each metric. Asterisks indicate that metrics are significantly different between groups (Table C1 in Appendix C).

**FIGURE 6:**
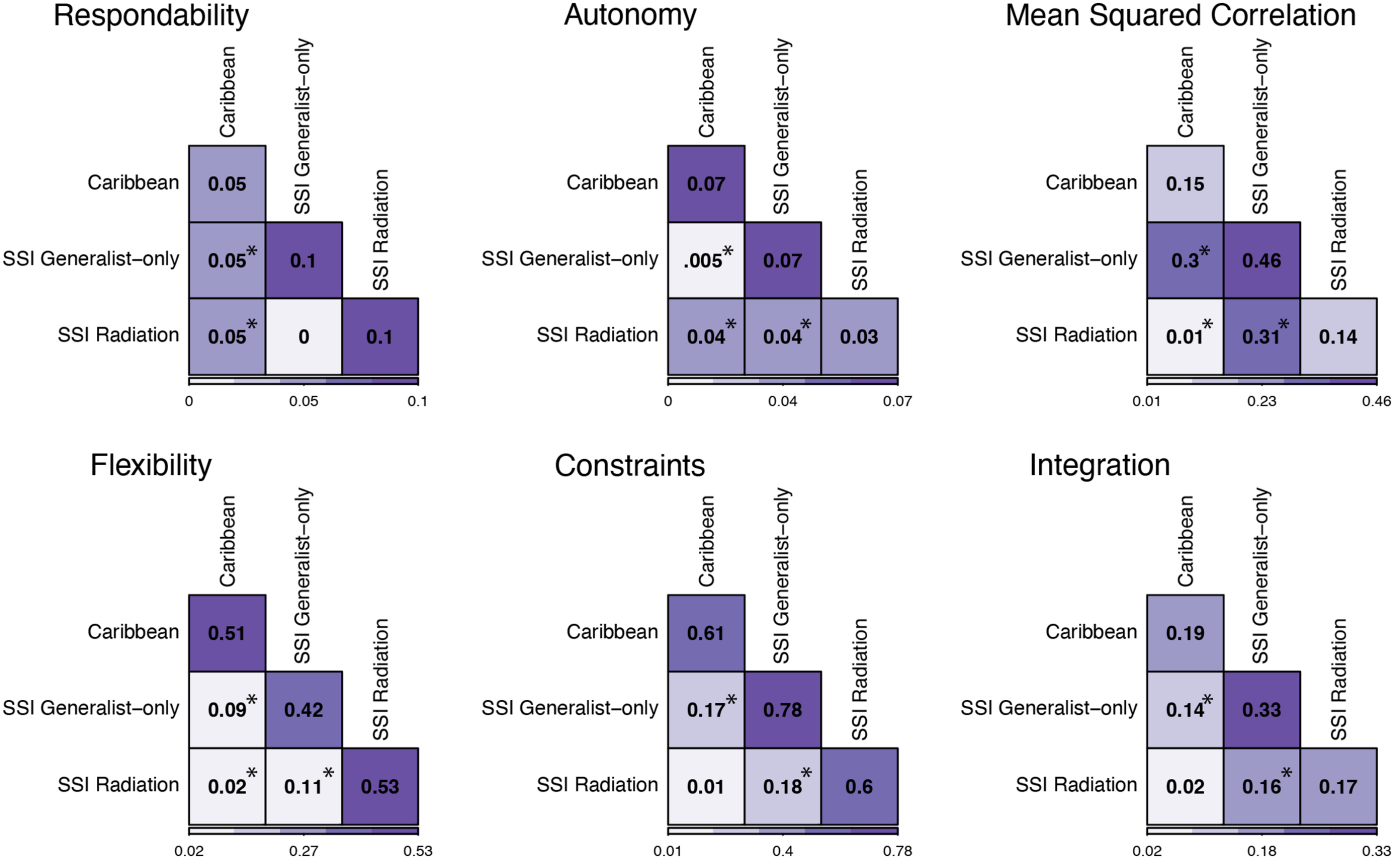
SSI generalist‐only populations have the lowest estimate of flexibility and the highest estimates of constraints, mean squared correlation, and integration. The squares along the longest diagonal show the mean values of the P matrix metrics for the corresponding groups. The other squares show the differences between means of groups. Fill colors represent low (light purple) and high (dark purple) values within each metric. Asterisks indicate significant differences between groups (Table C1 in Appendix C).

The visualization of the PCA suggested that the P matrix of SSI generalist‐only populations was distinct from both the Caribbean and SSI radiating groups (Figure 4). In direct comparisons, we found that the SSI radiating and Caribbean groups had the highest level of PCA similarity, indicating that the P matrices of these two populations were extremely similar (Figure 5; Table C1 in Appendix C). Direct comparisons of each of these groups with SSI generalist‐only populations also indicated that their P matrices were significantly different. Specifically, the P matrix of the SSI generalist‐only group had significantly higher levels of integration, constraints, and mean‐squared correlation between traits, with simultaneously lower estimates of flexibility than the P matrices of SSI radiating and Caribbean populations (Figure 6; Table C2 in Appendix C). In fact, there were only two P matrix features for which the SSI generalist‐only group was similar to other pupfish populations: respondability and autonomy. For respondability, both SSI radiating and SSI generalist‐only populations exhibited similar values (respondability = ~0.09), while the Caribbean population value was approximately half of this estimate (respondability = 0.048). On the other hand, autonomy estimates for the Caribbean and SSI generalist‐only groups were extremely similar (autonomy = ~0.07), while the P matrix of SSI radiating populations had significantly lower values (autonomy = 0.034) (Figure 6; Table C2 in Appendix C).

### 3.3 Differences in patterns of variation and covariation for specific traits

**FIGURE D2:**
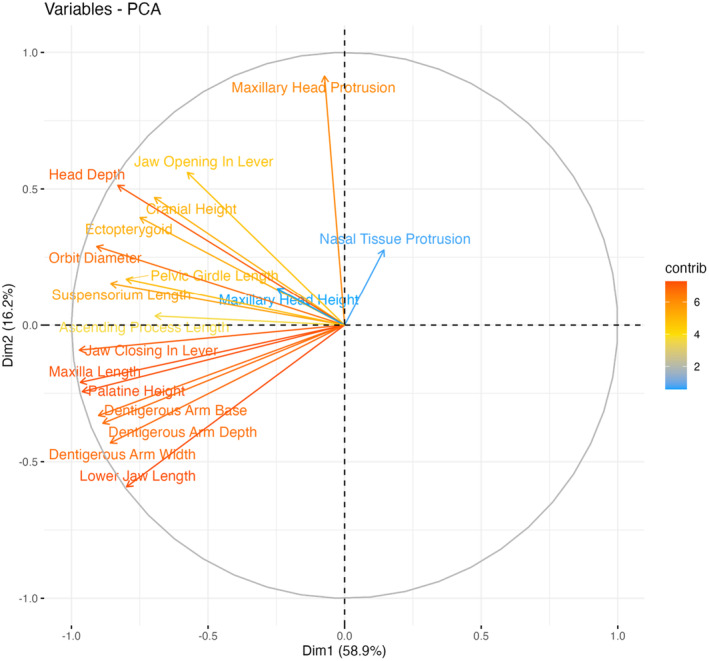
Visualization of the loadings for PC axes one and two from a principal components analysis calculated with the variance and covariance estimates for 18 craniofacial traits for the Caribbean, SSI generalist‐only, and SSI radiating populations. The colors of each loading represent their overall contribution (in percentages) to both axes.

The PCA investigating patterns across the P matrices of Caribbean, SSI generalist‐only, and SSI radiating groups indicated that jaw closing in‐lever, maxilla length, and palatine height had strong negative correlations with PC1 (~−0.96 for all three traits), while nasal tissue protrusion had the only positive correlation value for PC1 (0.014; Figure 4; Table D3, Figure D2 in Appendix D). For PC axis 2, we observed a strong positive correlation associated with maxillary head protrusion (0.91), moderate positive correlations for jaw opening in‐lever (0.56) and head depth (0.51), and moderate negative correlations for lower jaw length (−0.59) and dentigerous arm width (−0.43; Figure 4; Table D3, Figure D2 in Appendix D). Similar correlation values between traits along respective PC axes suggest that these traits share similar patterns of variation and covariation, while similar values with opposite signs indicate antagonistic patterns. Further investigation showed that most of the variation along PC1 was contributed by the SSI generalist‐only P matrix (52.5%), while most variation along PC2 was contributed by the SSI radiating P matrix (84.8%; Table D4 in Appendix D).

Along PC1, we saw that the major loadings of jaw closing in‐lever, maxilla length, and palatine height had a strong relationship in the negative direction with SSI generalist‐only traits, a variable relationship with SSI radiating traits, and a strong relationship in the positive direction with Caribbean traits (Figure 4; Table D4 in Appendix D). This suggests that as the covariation of jaw closing in‐lever, maxilla length, and palatine height increases, the covariation of other traits in SSI generalist‐only groups also increases, while the covariation in Caribbean groups decreases. The relationship is more complicated in SSI radiating groups as the covariation of some traits, such as lower jaw length and dentigerous arm width, had a positive relationship with the covariation of jaw closing in‐lever, maxilla length, and palatine height, while others, such as nasal tissue protrusion and maxillary head protrusion, had a negative relationship.

The major loadings for PC2 include maxillary head protrusion, lower jaw length, and jaw opening in‐lever (Table D3 in Appendix D). In general, the variation along PC2 corresponds to the SSI radiation P matrix (Figure 4; Table D4 in Appendix D). For instance, for the SSI radiating group, the covariation between maxillary head protrusion and jaw opening in‐lever had a positive relationship with one another but a negative relationship with lower jaw length, dentigerous arm width, and dentigerous arm base. These patterns were either significantly weaker, or opposite in the Caribbean and SSI generalist‐only matrices.

### 3.4 F2 hybrid crosses differ in their deviations from null expectations but exhibit similar average trait values

**FIGURE 7:**
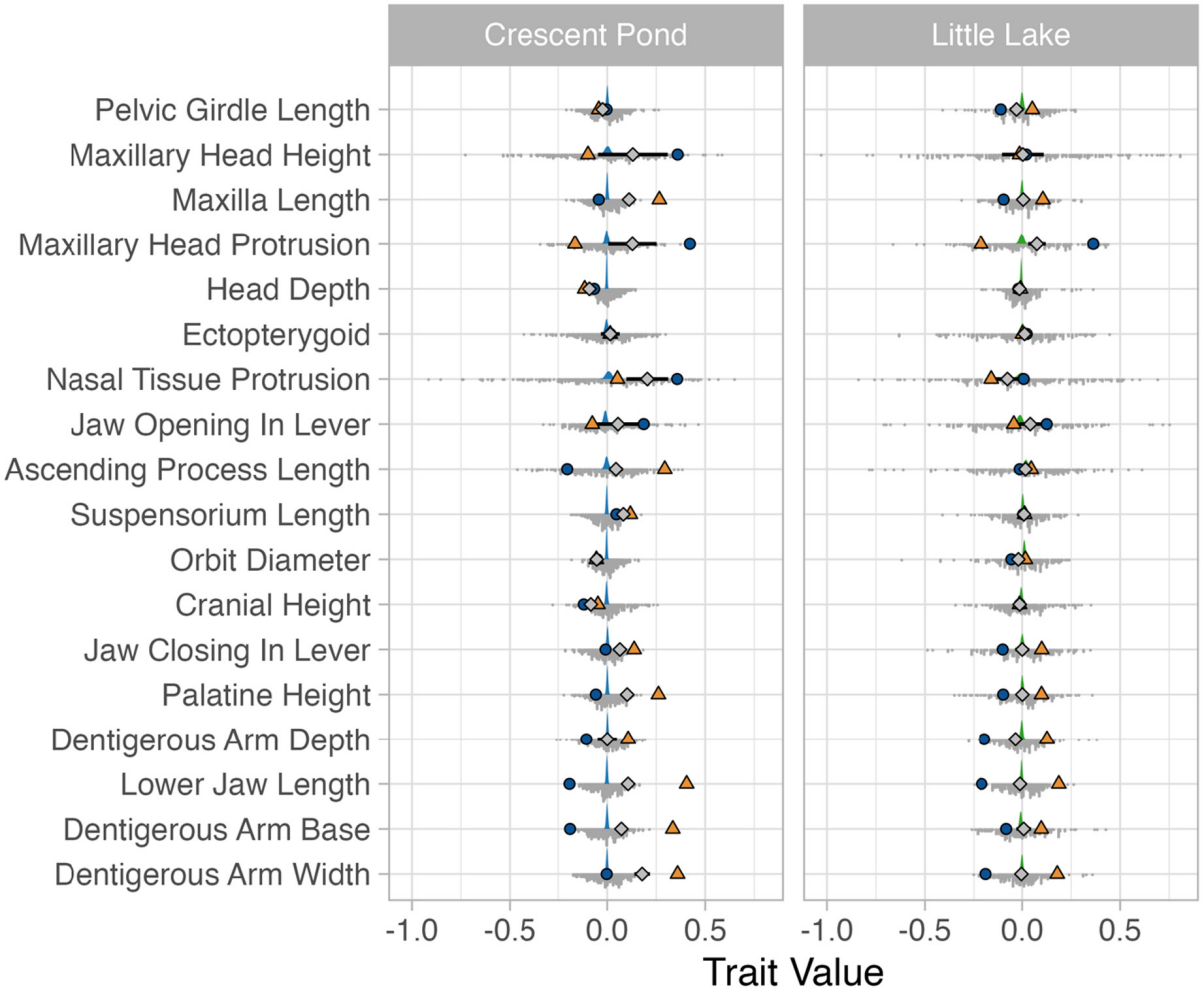
The estimated trait values of F2 hybrid pupfish (Snail‐eater ♂ X Scale‐eater ♀) compared to F0 parental species from Crescent Pond and Little Lake. Gray dots forming a histogram beneath each trait show the distribution of F2 phenotypes. The colored density graph above each histogram depicts the posterior draws from a Bayesian model with the 18 craniofacial trait values as the response variable and population as the fixed effect. Blue circles represent the F0 snail‐eater parental phenotype for each trait, while orange triangles represent the F0 scale‐eater parental phenotype for each trait. Finally, gray diamonds depict the mid‐parent trait value, which represents the expected phenotype trait value under the assumptions of additivity.

The Bayesian model results suggested that there was no difference in average trait values between Crescent Pond (mean trait value: −0.00096, 95% HDI: −0.0044, 0.0023) and Little Lake (mean trait value: 0.00032, 95% HDI: −0.0040, 0.0050) F2 hybrids. Estimates of 95% HDI for each of the 18 traits confirmed that there were no significant differences between F2 populations (Table E4 in Appendix E). Despite the lack of differentiation in trait values between these two populations, we found significantly different patterns of deviation from our null expectations between Crescent Pond and Little Lake. For example, the estimates for 16 of the 18 craniofacial traits in Little Lake met the assumptions of additivity, while only seven of the 18 traits in Crescent Pond met the same assumptions (Figure 7, Table E2 in Appendix E). Specifically, we found that estimates of cranial height and head depth were greater than expected in Crescent Pond along with estimates of orbit diameter for both F2 populations. Estimates of maxillary head protrusion were smaller than expected for Little Lake, and estimates of dentigerous arm width, dentigerous arm base, lower jaw length, palatine height, jaw closing in‐lever, suspensorium length, nasal tissue protrusion, and maxilla length were all smaller than expected in Crescent Pond (Figure 7).

### 3.5 Similarities and differences between Crescent Pond and Little Lake F2 hybrid P matrices

**FIGURE 8:**
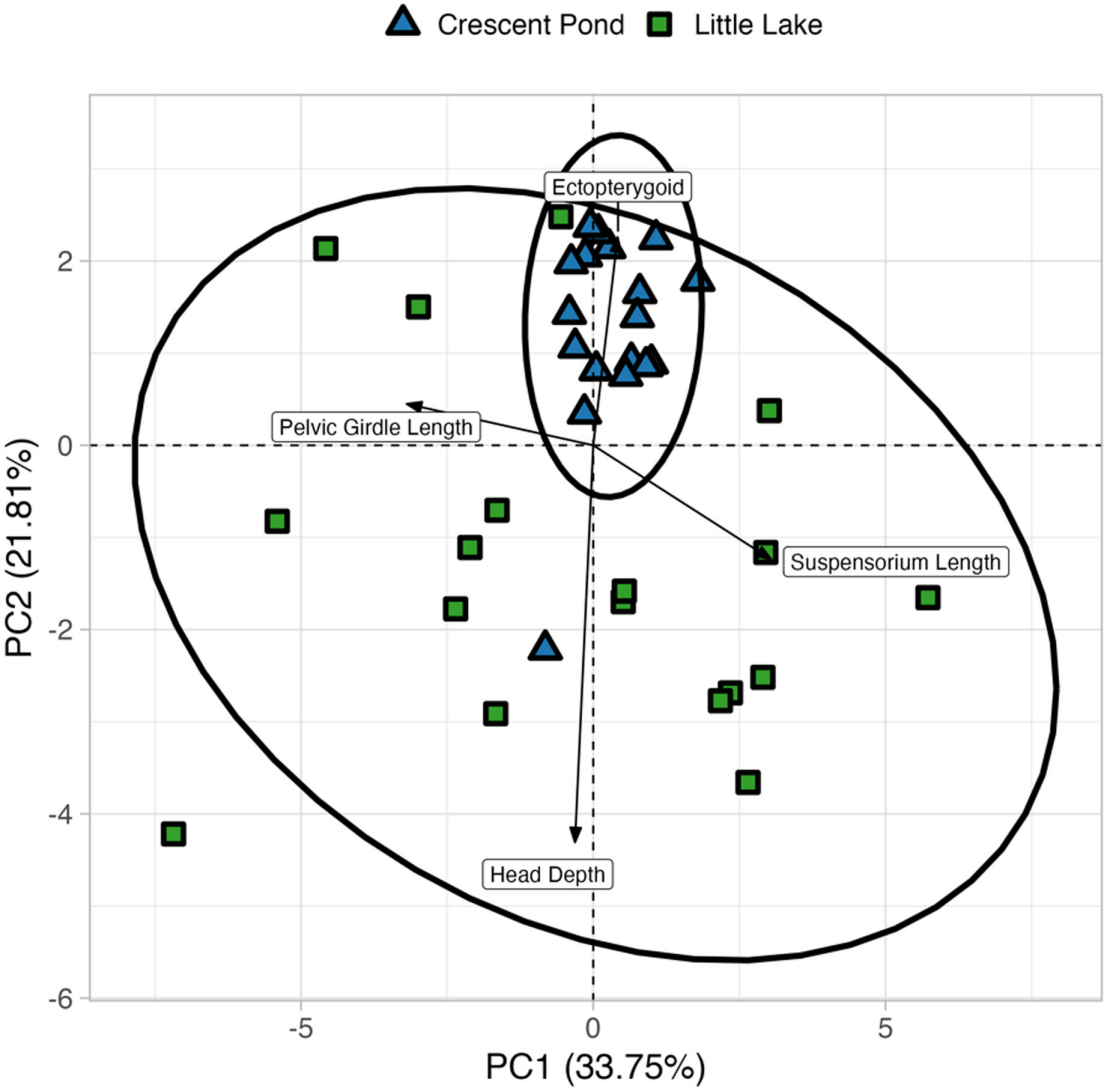
Results of the principal components analysis including the variance and covariance estimates for 18 craniofacial traits for F2 hybrids from Crescent Pond (blue triangles) and Little Lake (green squares). Colors represent species designations and ellipses represent 95% confidence intervals per group. Arrows display four craniofacial traits that are most strongly correlated with PC1 (pelvic girdle length and suspensorium length) and PC2 (ectopterygoid and head depth). Variation in Crescent Pond F2 hybrids aligns with PC2, while variation in Little Lake is spread across both axes.

The PCA visualization of F2 hybrid P matrices showed a large amount of overlap between populations, suggesting that their P matrices are quite similar (Figure 8). This interpretation is supported by a large estimate of PCA similarity between the two populations (~0.83) and the relative similarity in mean squared correlation, autonomy, and integration between populations (Tables C1 and C2 in Appendix C). Despite this overall appearance of similarity, there were still some distinct differences between populations. For instance, variation in the P matrix of Crescent Pond aligned very closely with PC axis 2, while variation in the Little Lake P matrix was uniformly distributed across PC1 and PC2 (Figure 8). Furthermore, we found smaller estimates of respondability (Crescent Pond: 0.022, Little Lake: 0.045) and flexibility (Crescent Pond: 0.58, Little Lake: 0.62) for the Crescent Pond P matrix compared to the Little Lake P matrix (Table C2 in Appendix C). Together, this suggests that there are unique relationships between traits in Crescent Pond and Little Lake.

### 3.6 Patterns of variance and covariance for specific traits

**FIGURE E1:**
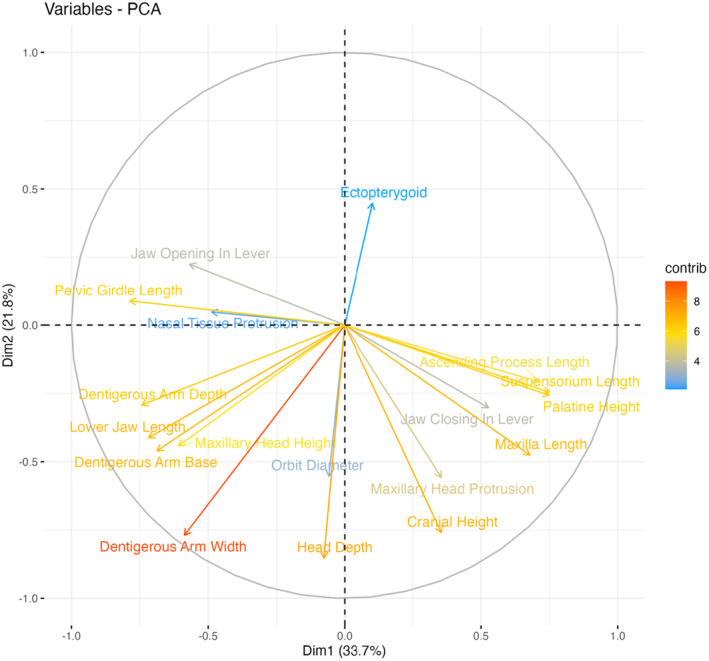
Visualization of the loadings for PC axes one and two from a principal components analysis calculated with the variance and covariance estimates for 18 craniofacial traits for F2 hybrids from Crescent Pond and Little Lake. The colors of each loading represent their overall contribution (in percentages) to both axes.

Suspensorium length, palatine height, and ascending process length had strong positive correlations with PC axis 1 (0.75, 0.75, and 0.71, respectively), while pelvic girdle length, dentigerous arm depth, and lower jaw length had strong negative correlations (−0.79, −0.74, −0.72 respectively; Table E5 in Appendix E). PC2 was defined by positive correlations with ectopterygoid (0.45) and jaw opening in‐lever (0.22) and strong negative correlations with head depth (−0.85), dentigerous arm width (−0.77), and cranial height (−0.76; Table E5 in Appendix E). Most variation across both axes could be attributed to patterns in the Little Lake P matrix (93% of variance across PC1 and 63% variance across PC2); however, we could still identify clear differences in the relationship between traits for each population. For instance, there was a strong positive relationship between covariation patterns of lower jaw length and jaw opening in‐lever in Little Lake, but this relationship was non‐existent in Crescent Pond (Figure 8; Table E6 in Appendix E). In fact, many relationships between traits captured by PC1 were strong in Little Lake but weak in Crescent Pond (Figure E1 in Appendix E). Maxillary head height was the only trait in Crescent Pond to show a strong positive relationship with head depth, dentigerous arm width, and cranial height, while several traits in Little Lake shared this relationship (Figure 8).

Finally, we found that overall variation within traits was similar between Crescent Pond and Little Lake (*T* test: *t* = −1.97, df = 27.45, *p*‐value = .059), but variances in Little Lake were greater than variances in Crescent Pond for all traits. Although similar between populations, only the variation in Crescent Pond met null expectations associated with the laws of independent assortment and segregation (expected variation: 0.14, observed variation: 0.013; *T* test: *t* = −0.14, df = 33.72, *p*‐value = .89). Variation in Little Lake was significantly higher than expected (expected variation: 0.007, observed variation: 0.029; *T* test: *t* = 3.05, df = 21.37, *p*‐value = .006). We found that covariation estimates between traits were not significantly different from one another (LM: *F* = 1.39, df = 17, *p*‐value = .13), but that overall Little Lake had higher estimates of covariation than Crescent Pond (LM: *F* = 30.1, df = 17, *p*‐value < .01).

The interaction between population and trait significantly affected regression coefficient values (LM: *F* = 1.93, df = 17, *p*‐value = .013). Within Crescent Pond, head depth, maxilla length, and palatine height had significantly higher regression coefficients than 50% of the measured traits. Within Little Lake, head depth, lower jaw length, and dentigerous arm width had higher regression coefficients than 50% of the measured traits. Comparing traits between populations, regression coefficients for orbit diameter (Tukey's HSD: *t* ratio = 2.67, *p*‐value = .0078), palatine height (Tukey's HSD: *t* ratio = 3.45, *p*‐value = .0078), jaw closing in‐lever (Tukey's HSD: *t* ratio = 2.22, *p*‐value = .027), and maxilla length (Tukey's HSD: *t* ratio = 2.18, *p*‐value = .003) were all higher in Crescent Pond than in Little Lake. Lastly, we found that squared correlation coefficients significantly varied between traits (LM: *F* = 10.19, df = 17, *p*‐value < .01), where dentigerous arm width, maxilla length, lower jaw length, and palatine height had higher correlation coefficients than 50% of traits in the data set.

## 4 DISCUSSION

### 4.1 Phenotypic covariation among traits significantly differs between the SSI radiating and non‐radiating groups

The first aim of this study was to investigate if unique multivariate phenotypes and covariation between traits were associated with pupfish diversification on San Salvador Island, Bahamas. While pupfish have radiated in several lakes on SSI, specialist species are notably absent in other lakes on the same and nearby islands, despite their close proximity and environmental similarity. We initially predicted that the pattern of diversification would be explained by shifts in the mean trait values and patterns of covariation in the SSI radiating group. Our comparisons between mean trait values support this prediction, as SSI radiating groups had larger trait values than SSI generalist‐only and Caribbean groups. This result was not surprising, as the hallmark traits of the specialist species within the radiation are the expanded dorsal head of the maxillae and the larger jaw apparatus (Hernandez et al., 2018; Martin & Wainwright, 2011).

Comparisons of the relationship between traits, however, suggest that shifts in patterns of covariation primarily occur in the SSI generalist‐only group, whereas the Caribbean and the SSI radiation have high levels of similarity between their P matrices (Figures 5 and 6). This could indicate that the P matrices of Caribbean populations of pupfish are “primed” for diversification but have not been exposed to the specific selective pressures that may induce such diversification. On the other hand, SSI generalist‐only populations are exposed to similar environmental pressures yet do not contain the full radiation, which may suggest that the phenotypic matrix structure for these populations may lack dimensions of phenotypic covariation that are necessary for diversification. Indeed, we found that the P matrices of these non‐radiating populations differed from those of SSI radiating and Caribbean populations (Figure 4), and our specific results indicate that diversification may be limited by stronger associations between traits, higher constraints, and lower flexibility (Figures 5 and 6).

### 4.2 Estimates of integration, mean trait correlation, constraints, and flexibility may limit pupfish diversification on San Salvador Island

Our results suggest that greater independence in trait variation is associated with diversification in this system. The P matrix of the SSI generalist‐only group had significantly greater estimates of integration and mean squared correlation between craniofacial traits, higher estimates of constraints, and lower estimates of flexibility – all measurements that can be interpreted as proxies for independence between traits (Table 2; Figure 6). For example, the SSI generalist‐only group had the highest levels of correlation between cranial traits, which ultimately may limit the phenotypic space that these populations can access (Goswami & Polly, 2010). Similarly, the constraint and flexibility estimates for the SSI generalist‐only group suggest that they are more limited in their ability to respond to and align with a wide range of selection gradients than the Caribbean or SSI radiating groups. This limited ability to respond to a range of selection gradients may explain why these populations are exposed to similar environmental conditions yet do not contain the snail‐ and/or scale‐eater specialists found in other lakes.

Other empirical studies also suggest that independence across many biological levels may be positively associated with diversification. Greater amounts of gene duplication, larger ratios of nonsynonymous:synonymous mutations, and lower levels of covariation between morphological traits have all been suggested as integral to diversification in some African cichlid radiations (Brawand et al., 2014; Machado et al., 2014; Selz et al., 2014) and represent independence at both the genetic and morphological levels. Similarly, Ravinet et al. (2014) suggest that populations of three‐spined stickleback (*Gasterosteus aculeatus*) from the Japan Sea have not colonized freshwater habitats and subsequently diversified, as many of the closely related Pacific freshwater populations have done, specifically due to the increased correlation between their morphology and dietary niche. Lastly, the flexible stem hypothesis suggests that the divergence within adaptive radiations emerges from plastic phenotypic variation in an ancestral population (West‐Eberhard, 2003). Through this lens, the properties of the Caribbean P matrix could be viewed as being plastic (i.e., low levels of constraints, high levels of flexibility, and low respondability)—estimates that remain largely the same in radiating groups but have shifted in non‐radiating groups. Other empirical studies, however, have found mixed results regarding the relationship between plasticity and diversification. Navalón et al. (2020) observed that higher integration, which may imply lower levels of plasticity, likely helped produce the diversity seen in the adaptive radiations of Darwin's finches and Hawaiian honeycreepers. On the other hand, lower levels of integration in brown trout (*Salmo trutta*) allowed them to diversify into a new ecological niche when faced with a new selective pressure (Závorka et al., 2017). These results support the idea that independence and flexibility, either within or between molecular, morphological, and/or behavioral traits, contribute to a group's ability to diversify. The findings presented in this study further support this conclusion.

### 4.3 Phylogenetic relationships between populations of pupfish

A caveat for the presented methods and results is that we did not correct for phylogenetic relationships when estimating or comparing P matrices across populations and species. Comparative methods are important tools for making comparisons between samples that violate assumptions of independence within comparison groups, such as comparing closely related species that vary in their evolutionary relatedness to each other. While the pupfish species and populations used in this study are indeed closely related, we would argue that the patterns and interpretations presented here are still informative.

Many previous studies estimate the evolutionary relationship between pupfish populations, and all suggest that SSI pupfishes form a monophyletic group that is sister to Caribbean pupfish populations (Martin, 2016; Martin & Feinstein, 2014; Richards et al., 2021; Richards & Martin, 2017). Based on this information, we may predict a high level of similarity in the P matrices of SSI generalist‐only and SSI radiating groups. On the other hand, these same studies suggest that the relationship between species varies across lakes on SSI, leading to two alternative predictions about P matrix similarity.

If the P matrices of pupfish species are fundamentally different from one another, we could expect a higher level of similarity between the SSI generalist‐only and Caribbean P matrices because the SSI radiating P matrix also contains trait values from two divergent species. We did not find any evidence, however, to suggest that the SSI radiating P matrix was biased toward specialist trait relationships over generalist relationships (Appendix F). If instead P matrices vary between populations, then we may expect Caribbean, SSI generalist‐only, and SSI radiation matrices to all differ from one another.

Regardless of which scenario most closely reflects reality, neither predicts the high level of similarity observed between Caribbean and SSI radiating P matrices. Furthermore, the fact that we detected differences between the SSI generalist‐only and Caribbean matrices indicates that this similarity is not a false positive driven by the close evolutionary relationship.

### 4.4 Which trait relationships may promote or inhibit diversification on San Salvador Island?

**FIGURE D3:**
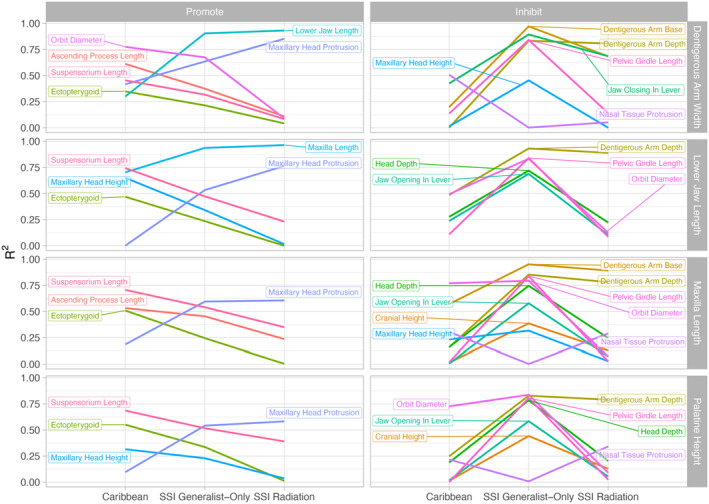
Visualization of the *R* ^2^ values from linear models investigating the relationship between the covariances of dentigerous arm width, lower jaw length, maxilla length, and palatine height and the traits labeled in each graph for Caribbean, SSI generalist‐only, and SSI radiating groups. The first column of graphs shows a relationship between traits that shift across groups in a manner that suggests that they promote diversification. The second column shows relationships between traits that may inhibit diversification, specifically in SSI generalist‐only groups, as they deviate from those seen in the Caribbean and SSI radiation.

Within the above phylogenetic context, parsimony predicts that unique trait relationships observed in the SSI radiating group may be associated with diversification. Indeed, we find that dentigerous arm width, lower jaw length, palatine height, and maxilla length – traits that have high correlations with one another in our PCA – display either increasing or decreasing strength in their relationships across pupfish groups, which supports this inference. To illustrate this pattern, we visualize the *R* ^2^ values from linear models with the covariation values of dentigerous arm width, lower jaw length, palatine height, or maxilla length as the response variable and the covariation values of all other traits as the predictor variable and display the relationships that may promote or inhibit divergence on SSI (Figure D3 in Appendix D). All four of the above traits have higher associations with suspensorium length and ectopterygoid in the Caribbean, weakened but intermediate relationships in the SSI generalist‐only group, and the weakest relationships in the SSI radiating group. This pattern could suggest that increased independence between these traits occurred during the initial colonization of SSI and further shifted during the radiating process.

Using a similar logic, we could also predict that unique trait relationships observed in the SSI generalist‐only group may hinder the diversification process. For instance, we find that the SSI generalist‐only group has a stronger relationship between dentigerous arm width, lower jaw length, palatine height, and maxilla length and (1) other jaw apparatus traits and (2) body size and shape traits compared to the Caribbean or SSI radiating groups (Figure D3 in Appendix D).

Dentigerous arm width, lower jaw length, palatine height, and maxilla length are traits with clear connections to feeding kinematics across many fish species (DeLaurier, 2019; Grubich & Westneat, 2006; Hulsey & García De León, 2005; Muñoz et al., 2018; Westneat, 2005). The similar patterns of covariance between these traits, along with their strong relationship to one another, suggest they may form a module in pupfishes. While all these traits seem to act together across groups, the SSI generalist‐only group is the only one that displays a strong relationship between this module and other traits such as head depth, maxillary head height, and jaw opening in‐lever (Figure D3 in Appendix D). This could mean that these additional relationships are constraining the jaw apparatus in SSI generalist‐only groups and preventing them from diversifying into additional ecological niches.

Overall, we observed that the P matrix of the generalist‐only group on SSI was distinct and generally more constrained than those of the Caribbean and SSI radiating groups, a pattern that could be explained by several mechanisms: First, this pattern may be due to founder effects (Barton & Charlesworth, 1984). For example, if the founding population of pupfish that first populated Wild Dilly Pond, Reckley Field Pond, Pain Pond, Moon Rock Pond, Six Pack Pond, and Mermaid Pond on SSI had more constrained P matrices than those that populated Crescent Pond and Little Lake, then the available variation may have been insufficient to produce the scale‐eating and/or snail‐eating phenotypes. Alternatively, the generalist‐only ponds may have once contained the full adaptive radiation, but the specialist species went extinct. We therefore may be observing differences in the P matrix attributes due to the shifting selective pressures that led to the extinction of specialists or observing P matrix patterns which are the result of selection post‐specialist extinction.

### 4.5 Inferences about the underlying mechanisms of craniofacial traits from F2 hybrid P‐matrices

In addition to making inferences about how variation in P matrices may contribute to diversification in the wild, this study also sought to investigate the underlying mechanisms associated with these traits on SSI by investigating the similarities and differences in P matrices and phenotype values of F2 hybrids from two separate crosses. We found that six of 18 traits met the null expectations of additivity in both populations (Figure 7), suggesting that the mechanisms underlying these traits generally fit the simple models often used in quantitative genetics, which assume Mendelian segregation, independent assortment, and the presence of additive genetic effects (i.e., no dominance or epistatic effects; Falconer, 1996; Roff, 1997). Of the remaining 12 traits, we found that only orbit diameter failed to meet additive expectations in both crosses. Crescent Pond and Little Lake F2 hybrids both exhibited larger mean estimates of this trait than additive expectations, a pattern that could be produced through dominance or epistatic effects. Ten of the remaining traits met null expectations of additivity in Little Lake but not in Crescent Pond, and one trait, maxillary head protrusion, met expectations in Crescent Pond but not in Little Lake. This pattern could again be driven by dominance or epistatic effects, which are likely different between ponds; however, it may also be driven by other factors such as new mutations in F2 hybrids, recessive alleles, lethality of specific allele combinations (i.e., Dobzhansky–Muller or constitutive incompatibilities), or gene drive (Andersen & Rockman, 2022; Burkart‐Waco et al., 2012; Chevin et al., 2014; Rick & Smith, 1953). There is indeed some evidence for this in our current dataset. For example, many traits, especially in Little Lake, have much higher levels of variation in phenotype trait values and exhibit phenotypes that fall well outside those observed in the F0 parental types. Little Lake also shows higher levels of covariation between traits than we observed in Crescent Pond. The increased variation in trait values in both ponds may imply that new combinations of alleles or epistatic effects are responsible for this additional variation, while the high levels of covariation in Little Lake may suggest that these traits share a common mechanism that is not present in the Crescent Pond cross.

Increased variation and covariation within and between hybrid traits is commonly observed in nature, and there is even evidence that hybridization may drive speciation events in many systems including pupfish (Bell & Travis, 2005; Richards et al., 2019, 2021; Seehausen, 2004; Selz et al., 2014). On the other hand, F2 hybrids in Crescent Pond do not display phenotype values that meet or exceed the scale‐eater parental values for traits such as palatine height, lower jaw length, and dentigerous arm width (Figure 7), implying that dominance, segregation distortion, or lethality may be at play. While lethal combinations of alleles are possible, it is more commonly expected to occur when species undergo divergence for longer periods of time than what is observed in the pupfish system (Coyne & Orr, 1997). Therefore, future work needs to (1) identify the loci associated with these traits and (2) investigate whether these loci suffer from segregation distortion as a first step to untangling the underlying mechanisms of these traits.

### 4.6 What can the F2 hybrid P matrix tell us about craniofacial traits in SSI?

In this study, we include calculations of the regression coefficient and the squared correlation coefficient between traits. The regression coefficients calculated in this study describe how much change is observed in Trait B when Trait A increases by one unit. Values close to 0 suggest that Trait A has little effect on Trait B; values close to positive or negative one suggest that the magnitude of change A and B are similar; and values outside of the negative to positive one range suggest that change in Trait B exceeds that of Trait A (although the sign dictates if the change is in the same direction as Trait A; Kelly, 2009). The formula for squared correlation coefficients is often used between genetic loci to calculate the strength of association between alleles (i.e., *r* ^2^ as a measure of linkage disequilibrium; VanLiere & Rosenberg, 2008). In the context of this study, high values suggest that individuals with large trait values for Trait A also have large values for Trait B, and vice versa. Interpreting the patterns of both estimates together may give us insight into the traits that are integral to producing the unique craniofacial pupfish traits found on SSI. For instance, head depth in both Little Lake and Crescent Pond produced the largest amount of change in other traits, which could mean that the underlying mechanisms responsible for variation in this trait are reused across most other craniofacial traits in pupfishes. Maxilla length and palatine height in Crescent Pond and lower jaw length and dentigerous arm width in Little Lake also produced large changes in other traits, further supporting this idea. The average squared correlation coefficients were also significantly higher for these traits compared to most of the remaining traits in the dataset. From a mechanistic view point, this pattern may reflect high levels of linkage disequilibrium or it may suggest that these traits utilize a shared underlying mechanism, either through pleiotropy or shared genetic or developmental pathways. Previous work in pupfishes has found differential gene expression and fixed single‐nucleotide polymorphism differences between species for regions of the genome associated with the wnt signaling pathway, which has ties to craniofacial diversity across a wide array of organisms (Lencer et al., 2017; Lencer & Mccune, 2020; Richards & Martin, 2022). While much more work is needed to determine if this specific pathway produces the observed craniofacial diversity on SSI, these previous results support at least the possibility of the reuse of a conserved evolutionary pathway as a mechanism for producing pupfish diversity.

## 5 CONCLUSION

In this study, we sought to more fully understand which factors contribute to diversity in the *Cyprinodon* pupfish system. Our data support the idea that phenotypic variation and covariation, or the relationship between traits, is an additional and important axis of variation that contributes to diversification in the pupfish system. When investigating the factors driving diversification between pupfish groups, we find that the lack of a flexible P matrix may impede the ability to diversify. We also used F2 hybrid P matrices to make inferences about underlying mechanisms that produce variation in craniofacial traits in the pupfish system. We found that many traits adhere to the expectations of a simple additive genetic model. Yet, key traits such as head depth, maxilla length, palatine height, lower jaw length, and dentigerous arm width appear to be influenced by nonadditive genetic effects and produce large phenotypic changes in other traits. Future work should examine whether this relationship between flexibility and diversification is generalizable across many species or is specific to *Cyprinodon* pupfish.

## AUTHOR CONTRIBUTIONS

**Julia C. Dunker:** Conceptualization (equal); data curation (equal); formal analysis (equal); investigation (equal); methodology (equal); visualization (equal); writing – original draft (equal); writing – review and editing (equal). **Michelle E. St. John:** Conceptualization (equal); data curation (equal); formal analysis (equal); investigation (equal); methodology (equal); project administration (lead); visualization (equal); writing – original draft (equal); writing – review and editing (equal). **Christopher H. Martin:** Conceptualization (equal); data curation (equal); funding acquisition (lead); writing – review and editing (equal).

## CONFLICT OF INTEREST STATEMENT

The authors declare no conflict of interest.

### OPEN RESEARCH BADGES

This article has earned an Open Data badge for making publicly available the digitally‐shareable data necessary to reproduce the reported results. The data is available at https://osf.io/6e4wx/.

## Figures and Tables

**FIGURE A1:**
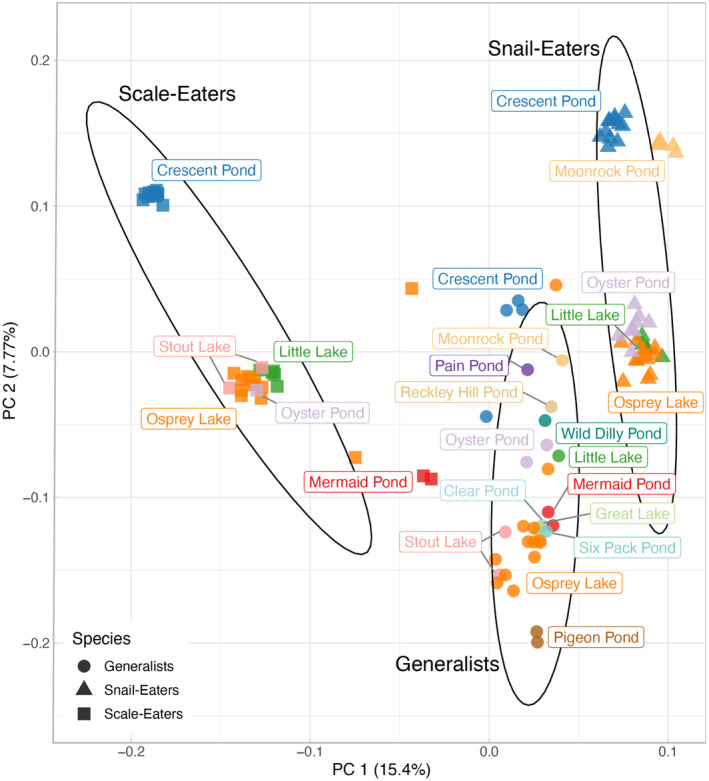
Results of a principal component analysis investigating the genetic relationships between generalist, snail‐eater, and scale‐eater pupfish species in lakes across San Salvador Island, Bahamas. Points represent sequenced individuals (*n* = 110), shapes represent species designations based on morphological traits of each specimen (generalists: circles, snail‐eaters: triangles, scale‐eaters: squares), colors, and labels correspond to 14 lakes on SSI that were sampled for this dataset, and ellipses represent 95% confidence intervals per group. Figure modified from Richards et al. (2021).

**FIGURE B1:**
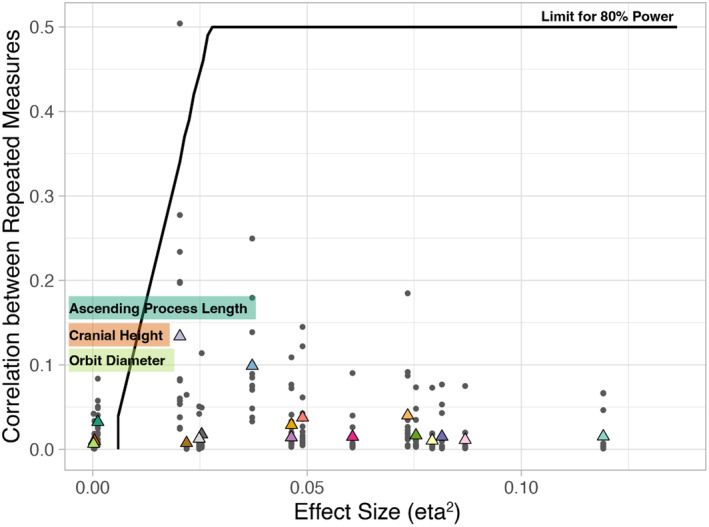
Visualization of the results of power analyses for a one‐way repeated measures analysis of variance. We used a range of different correlations, effect sizes, and numbers of individuals within each population to estimate the total sample sizes necessary to detect differences between groups with 80% power. The black line indicates the cutoff where the sample size of this dataset (*n* = 188) is sufficient for detecting differences between groups given the observed correlation between traits and the estimated effect size. Colored triangles represent the mean correlation for each trait, while gray dots show the correlations for each trait combination.

**FIGURE B2:**
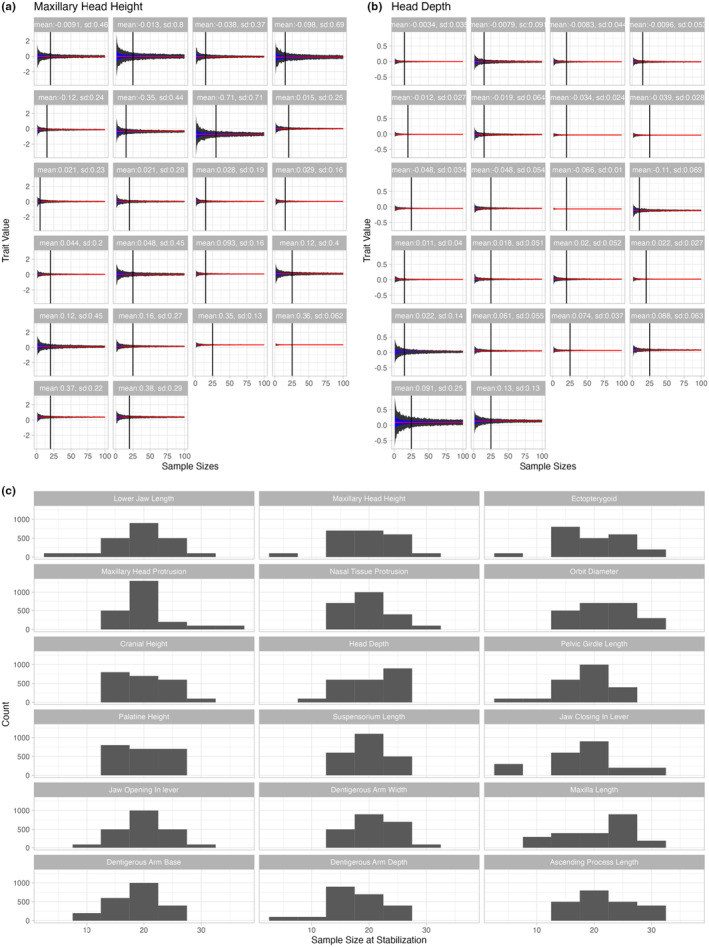
Visualization of Monte Carlo simulations investigating how sample size affects estimates of variation. Panels a and b display samples of simulations using observed means and standard deviation observed for maxillary head height and head depth in our dataset. The red line in each panel represents the mean trait values at each sample size. The black ribbons show the minimum and maximum trait value at each sample size, and the blue ribbons show the standard deviation around the mean at each sample size. The vertical line in each graph indicates where increasing sample size no longer significantly impacts estimates of standard deviation (i.e., where estimates of variation stabilize). Panel c shows histograms of the sample sizes associated with stabilization across traits.

## Data Availability

Upon acceptance, data and scripts will be posted on GitHub. For submission and review, data and scripts can be viewed here: https://github.com/stjohn3/Ecology_Evolution_Submision.

## APPENDIX A POPULATION GROUPINGS

For comparisons, we formed three focal groups: SSI generalist‐only, SSI radiating, and the Caribbean. The PCA (Figure A1) indicates that generalists, snail‐eaters, and scale‐eaters on SSI form distinct clusters based on their genetic variation. The PCA visualization also suggests that: (1) Oyster Pond scale‐eaters and generalists fall well within the 95% confidence intervals for their species designations, justifying their inclusion in the SSI radiating population analyses and (2) Mermaid Pond “scale‐eaters” (designated as such based on their morphological traits) do not fall within the 95% confidence interval for genetically clustering scale‐eaters, and are instead closer to the centroid of generalists. Although previous studies suggest that Mermaid Pond contains scale‐eating individuals (Martin & Wainwright, 2013a), more recent work suggests that these individuals are distinct from scale‐eaters (Richards et al., 2021. Richards & Martin, 2022). Furthermore, there are no published sightings of snail‐eating pupfish in Mermaid Pond. For these reasons, we included Mermaid Pond in the generalist‐only population calculations as opposed to the SSI radiating populations. Finally, snail‐eaters have previously been documented in Moon Rock and Wild Dilly Pond, but scale‐eating specialists have never been found in these locations (Martin & Feinstein, 2014). Considering that we did not have access to any of the snail‐eater specimens from these locations and that these populations have never contained the full radiation of pupfish, we included Moon Rock and Wild Dilly Pond in the generalist‐only population calculations as opposed to the SSI radiating populations.

## APPENDIX B POWER ANALYSES AND MONTE CARLO SIMULATIONS

To support our claim that sample sizes are sufficient for detecting differences between the groups of interest (i.e., SSI radiation, SSI generalist‐only, and Caribbean), we include the results of power analyses, which calculate the required minimum sample size across the range of effect sizes (eta2: 0.0002–0.12), the number of individuals per population (*n* = 7–21), and the correlations of trait estimates for individuals observed in our dataset (corr: 0.00046–0.50). From these iterative power analyses, we were able to determine for which combination of our parameters we would have sufficient sample sizes to detect differences between groups with 80% power. We compared these cutoffs to the observed values for each trait and population (Figure B1). Ultimately, we found that we have enough samples to detect differences between groups for all traits except for ascending process length, orbit diameter, and cranial height.

We also performed Monte Carlo simulations to investigate at which sample sizes estimates of variation stabilize based on observed means and standard deviations of traits from our dataset. To perform these simulations, we first calculated the average trait values (range: −0.71 to 0.42) and standard deviations (range: 0.01–0.8) for each trait across the potential groups of our data set (19 groups representing unique species per population, plus the additional three parameter estimates associated with Caribbean, SSI generalist‐only, and SSI radiating groups). We used these observed means and standard deviations to generate normal distributions to represent hypothetical populations to sample from. We resampled from these distributions using sample sizes from 1 to 100 for 1000 iterations, recalculating the mean each time. For each of our 22 groups, we ended up with 1,800,000 independent estimates of means across the sample size range of 1–100. At each sample size, we then calculated the standard deviation as a metric representing the amount of variation introduced due to the sample size. As expected, as sample size increased, the standard deviation decreased; however, the amount of variation at low sample sizes was dependent on the original parameters of the dataset (Figure B2a,b).

To determine at which sample sizes we observed a stabilization of variation (i.e., when adding more samples did not significantly affect the estimates of SD), we grouped sample sizes into sets of 5 (e.g., 1–5, 6–10, 11–15, etc.) and calculated the derivatives for each of these groups across the sampling range. We used a one‐sample *t*‐test to determine at which sampling range the derivative no longer significantly differed from zero using a conservative cutoff of nonsignificance with *p* > .1. Here, a derivative of zero represents when variation in SD is no longer significantly affected by the addition of more samples (i.e., when variation is stable). We determined at which sample size we first observed non‐deviance from zero for each of the observed parameters from our dataset and visualized these results using a histogram (Figure B2c) Overall, we found that the median sample size where stabilization occurred was at 20 individuals (range: 5–35 individuals; Figure B2c).

## APPENDIX C P MATRIX STATISTICAL ANALYSES

**TABLE C1:** PCA similarity estimates, including upper and lower 95% confidence intervals (bootstrapping; iterations = 100).

Population	PCA similarity
SSI Radiation/Caribbean	0.71 (0.69, 0.72)
SSI Generalist‐only/Caribbean	0.61 (0.59, 0.62)
SSI Generalist‐only/SSI Radiation	0.6 (0.59, 0.62)
CP Hybrid/LL Hybrid	0.83 (0.83, 0.84)

*Note*: The PCA similarity metrics estimate the similarity between two matrices, ranging from 0 to 1.

**TABLE C2:** Mean matrix statistics estimates, including upper and lower 95% confidence intervals (bootstrapping; iterations = 100), for focal groups.

Population	Respondability	Autonomy	Mean squared correlation	Flexibility	Constraints	Integration
Caribbean	0.048 (0.046, 0.049)	0.07 (0.068, 0.072)	0.15 (0.15, 0.16)	0.51 (0.51, 0.52)	0.61 (0.6, 0.62)	0.19 (0.19, 0.2)
SSI Generalist‐only	0.095 (0.09, 0.1)	0.075 (0.073, 0.078)	0.46 (0.44, 0.47)	0.42 (0.42, 0.43)	0.78 (0.77, 0.79)	0.33 (0.32, 0.34)
SSI Radiation	0.099 (0.095, 0.1)	0.034 (0.033, 0.036)	0.14 (0.14, 0.15)	0.53 (0.52, 0.53)	0.6 (0.6, 0.62)	0.17 (0.16, 0.17)
CRP Hybrid	0.022 (0.022, 0.022)	0.15 (0.15, 0.15)	0.11 (0.11, 0.11)	0.58 (0.58, 0.59)	0.56 (0.56, 0.57)	0.13 (0.13, 0.13)
LL Hybrid	0.045 (0.045, 0.046)	0.14 (0.14, 0.14)	0.1 (0.098, 0.1)	0.62 (0.62, 0.62)	0.54 (0.54, 0.55)	0.1 (0.1, 0.1)

*Note*: Each of the six statistics represents a descriptive characteristic of the P matrix for the indicated group. A full description of each statistic can be found in Table 2 of the main text.

## APPENDIX D ESTIMATES FOR CARIBBEAN, SSI GENERALIST‐ONLY, AND SSI RADIATING GROUP ANALYSES

**TABLE D1:** Estimated marginal mean trait values and 95% HDI for Caribbean, SSI generalist only, and SSI radiating groups from a Bayesian mixed effects model with the 18 craniofacial trait values as the response variable, pupfish group and species designations as fixed effects, and population ID as a random effect.

Trait	Caribbean	SSI generalist‐only	SSI radiation
Dentigerous arm width	−0.075 (−0.16, 0.007)	−0.076 (−0.16, 0.00044)	0.076 (−0.014, 0.17)
Dentigerous arm base	−0.081 (−0.13, −0.025)	−0.067 (−0.13, −0.015)	0.078 (0.035, 0.12)
Lower jaw length	−0.14 (−0.18, −0.084)	−0.095 (−0.14, −0.049)	0.062 (0.023, 0.11)
Dentigerous arm depth	−0.16 (−0.23, −0.091)	−0.13 (−0.19, −0.062)	0.061 (−0.00067, 0.12)
Palatine height	−0.067 (−0.12, −0.018)	−0.054 (−0.1, −0.0024)	0.079 (0.03, 0.12)
Jaw closing in lever	−0.04 (−0.11, 0.037)	−0.063 (−0.13, 0.0086)	0.077 (0.0056, 0.14)
Cranial height	−0.12 (−0.17, −0.065)	−0.14 (−0.19, −0.083)	0.015 (−0.021, 0.055)
Orbit diameter	−0.1 (−0.15, −0.057)	−0.11 (−0.15, −0.064)	0.0025 (−0.043, 0.052)
Suspensorium length	0.0014 (−0.083, 0.087)	−0.021 (−0.094, 0.063)	0.086 (0.0039, 0.18)
Ascending process length	−0.049 (−0.17, 0.074)	−0.073 (−0.18, 0.051)	0.011 (−0.1, 0.12)
Jaw opening in lever	−0.13 (−0.24, −0.012)	−0.072 (−0.18, 0.041)	0.15 (0.059, 0.25)
Nasal tissue protrusion	−0.027 (−0.29, 0.22)	0.077 (−0.16, 0.32)	−0.06 (−0.29, 0.19)
Ectopterygoid	−0.11 (−0.22, 0.0047)	−0.048 (−0.15, 0.066)	0.076 (−0.025, 0.18)
Head depth	−0.11 (−0.15, −0.052)	−0.094 (−0.14, −0.045)	0.0015 (−0.053, 0.057)
Maxillary head protrusion	−0.089 (−0.19, 0.021)	−0.026 (−0.13, 0.074)	0.14 (0.043, 0.24)
Maxilla length	−0.064 (−0.11, −0.012)	−0.061 (−0.11, −0.013)	0.076 (0.032, 0.12)
Maxillary head height	−0.18 (−0.51, 0.1)	−0.2 (−0.49, 0.091)	0.19 (−0.11, 0.51)
Pelvic girdle length	−0.057 (−0.13, 0.0099)	−0.055 (−0.12, 0.0095)	−0.027 (−0.085, 0.03)

**TABLE D2:** Estimated marginal mean trait values and 95% HDI for generalist, snail‐eater, and scale‐eater pupfish species from a Bayesian mixed effects model with the 18 craniofacial trait values as the response variable, pupfish group and species designations as fixed effects, and population ID as a random effect.

Trait	Generalists	Snail‐eaters	Scale‐eaters
Dentigerous arm width	0.041 (−0.0044, 0.087)	−0.22 (−0.33, −0.13)	0.11 (0.032, 0.19)
Dentigerous arm base	0.038 (0.014, 0.063)	−0.21 (−0.3, −0.13)	0.11 (0.041, 0.17)
Lower jaw length	0.037 (0.014, 0.06)	−0.34 (−0.41, −0.27)	0.13 (0.08, 0.19)
Dentigerous arm depth	0.056 (0.023, 0.089)	−0.28 (−0.36, −0.18)	−0.0061 (−0.081, 0.06)
Palatine height	0.034 (0.0092, 0.058)	−0.17 (−0.25, −0.1)	0.094 (0.039, 0.15)
Jaw closing in lever	0.041 (0.0056, 0.078)	−0.11 (−0.21, −0.0032)	0.042 (−0.036, 0.12)
Cranial height	0.053 (0.033, 0.077)	−0.15 (−0.23, −0.064)	−0.14 (−0.2, −0.083)
Orbit diameter	0.036 (0.011, 0.06)	−0.14 (−0.2, −0.074)	−0.11 (−0.16, −0.062)
Suspensorium length	0.0089 (−0.034, 0.054)	−0.024 (−0.12, 0.082)	0.082 (0.0034, 0.18)
Ascending process length	0.039 (−0.022, 0.097)	−0.17 (−0.34, 0.0085)	0.016 (−0.12, 0.15)
Jaw opening in lever	0.038 (−0.015, 0.089)	−0.024 (−0.19, 0.15)	−0.064 (−0.19, 0.06)
Nasal tissue protrusion	−0.036 (−0.17, 0.083)	0.18 (−0.18, 0.52)	−0.15 (−0.42, 0.094)
Ectopterygoid	0.051 (−0.004, 0.1)	−0.098 (−0.25, 0.064)	−0.032 (−0.16, 0.077)
Head depth	0.038 (0.011, 0.068)	−0.1 (−0.17, −0.044)	−0.13 (−0.18, −0.085)
Maxillary head protrusion	0.056 (0.0058, 0.11)	0.24 (0.097, 0.39)	−0.27 (−0.38, −0.16)
Maxilla length	0.04 (0.016, 0.064)	−0.17 (−0.24, −0.095)	0.078 (0.022, 0.13)
Maxillary head height	0.093 (−0.072, 0.25)	−0.072 (−0.45, 0.27)	−0.21 (−0.51, 0.06)
Pelvic girdle length	0.031 (0.0022, 0.064)	−0.086 (−0.19, 0.019)	−0.084 (−0.16, −0.013)

**TABLE D3:** Correlation of loadings with PC axes one and two from a principal components analysis calculated with the variance and covariance estimates for 18 craniofacial traits for the Caribbean, SSI generalist‐only, and SSI radiating populations.

Trait	PC1	PC2
Dentigerous arm width	−0.86	−0.43
Dentigerous arm base	−0.90	−0.33
Lower jaw length	−0.80	−0.59
Dentigerous arm depth	−0.89	−0.36
Palatine height	−0.96	−0.24
Jaw closing in lever	−0.97	−0.091
Cranial height	−0.70	0.47
Orbit diameter	−0.91	0.29
Suspensorium length	−0.86	0.15
Ascending process length	−0.69	0.034
Jaw opening in lever	−0.58	0.56
Nasal tissue protrusion	0.14	0.28
Ectopterygoid	−0.75	0.40
Head depth	−0.83	0.51
Maxillary head protrusion	−0.074	0.91
Maxilla length	−0.97	−0.21
Maxillary head height	−0.25	0.13
Pelvic girdle length	−0.80	0.17

**TABLE D4:** Contribution of the variation present in the P matrices of the Caribbean, SSI generalist‐only, and SSI radiating groups to PC axes one and two.

Caribbean			SSI generalist‐only			SSI radiation		
Trait	PC1	PC2	Trait	PC1	PC2	Trait	PC1	PC2
Dentigerous arm depth	1.16	0.216	Jaw opening in lever	7.63	1.78	Maxillary head protrusion	7.57	28.5
Jaw closing in lever	0.773	0.186	Ectopterygoid	4.17	1.19	Lower jaw length	0.759	15.2
Lower jaw length	0.839	0.177	Jaw closing in lever	6.23	1.03	Dentigerous arm width	0.219	10.0
Palatine height	0.770	0.145	Suspensorium length	2.56	0.951	Dentigerous arm base	0.172	6.47
Jaw opening in lever	1.42	0.145	Palatine height	3.35	0.706	Dentigerous arm depth	0.203	5.17
Dentigerous arm base	0.620	0.138	Maxilla length	3.22	0.671	Palatine height	0.0211	4.72
Maxilla length	0.835	0.131	Maxillary head protrusion	1.47	0.657	Maxilla length	0.00304	4.17
Pelvic girdle length	1.78	0.112	Dentigerous arm base	3.04	0.608	Jaw opening in lever	1.69	3.40
Suspensorium length	0.681	0.106	Head depth	1.62	0.595	Jaw closing in lever	0.0232	2.75
Orbit diameter	0.931	0.0824	Maxillary head height	7.01	0.574	Nasal tissue protrusion	9.46	1.21
Dentigerous arm width	0.306	0.0706	Ascending process length	2.40	0.568	Ascending process length	0.0592	0.915
Nasal tissue protrusion	2.85	0.0664	Dentigerous arm width	3.49	0.535	Suspensorium length	0.569	0.882
Head depth	1.34	0.0587	Dentigerous arm depth	2.60	0.447	Cranial height	0.423	0.510
Maxillary head height	4.18	0.0459	Lower Jaw length	1.88	0.377	Ectopterygoid	0.149	0.442
Ectopterygoid	0.111	0.0203	Cranial height	0.192	0.330	Head depth	0.691	0.368
Cranial height	1.49	0.0104	Pelvic girdle length	1.02	0.315	Pelvic girdle length	0.375	0.0599
Ascending process length	0.375	0.00276	Orbit diameter	0.435	0.198	Orbit diameter	0.513	0.00618
Maxillary head protrusion	1.11	0.0000133	Nasal tissue protrusion	0.147	0.0893	Maxillary head height	1.22	0.00372
Total variation	21.6%	1.71%		52.5%	11.6%		24.1	84.8%

## APPENDIX E ESTIMATES FOR F2 HYBRID ANALYSES

**TABLE E1:** Trait values for F0 snail‐eater and scale‐eater parental types for the Crescent Pond and Little Lake crosses.

Trait	Crescent pond	Crescent pond	Little Lake	Little Lake
	Scale‐eater	Snail‐eater	Scale‐eater	Snail‐eater
Dentigerous arm width	0.36	−0.0024	0.18	−0.19
Dentigerous arm base	0.33	−0.19	0.098	−0.081
Lower jaw length	0.41	−0.19	0.19	−0.21
Dentigerous arm depth	0.11	−0.11	0.13	−0.19
Palatine height	0.26	−0.058	0.099	−0.097
Jaw closing in lever	0.14	−0.0081	0.10	−0.099
Cranial height	−0.047	−0.12	−0.013	−0.012
Orbit diameter	−0.055	−0.051	0.017	−0.055
Suspensorium length	0.12	0.047	0.013	0.0054
Ascending process length	0.29	−0.20	0.046	−0.012
Jaw opening in lever	−0.076	0.19	−0.042	0.13
Nasal tissue protrusion	0.052	0.36	−0.16	0.0080
Ectopterygoid	0.015	0.016	0.0017	0.023
Head depth	−0.11	−0.066	−0.0083	−0.019
Maxillary head protrusion	−0.16	0.42	−0.21	0.36
Maxilla length	0.27	−0.043	0.11	−0.095
Maxillary head height	−0.098	0.36	−0.013	0.021
Pelvic girdle length	−0.043	−0.0032	0.052	−0.11

**TABLE E2:** Average trait value estimates for Crescent Pond and Little Lake F2 hybrids under the assumptions of additivity.

Trait	Crescent pond F2 additive expectations	Little Lake F2 additive expectations
Dentigerous arm width	0.18	−0.0038
Dentigerous arm base	0.073	0.0081
Lower jaw length	0.11	−0.011
Dentigerous arm depth	0.00054	−0.034
Palatine height	0.10	0.0012
Jaw closing in lever	0.065	0.00044
Cranial height	−0.083	−0.012
Orbit diameter	−0.053	−0.019
Suspensorium length	0.083	0.0091
Ascending process length	0.045	0.017
Jaw opening in lever	0.055	0.042
Nasal tissue protrusion	0.20	−0.075
Ectopterygoid	0.016	0.013
Head depth	−0.090	−0.014
Maxillary head protrusion	0.13	0.076
Maxilla length	0.11	0.0057
Maxillary head height	0.13	0.0037
Pelvic girdle length	−0.023	−0.029

*Note*: Estimates are the mid parent values calculated as (Trait Value_Parent1_ + Trait Value_Parent2_)/2.

**TABLE E3:** Estimates of expected variation for Crescent Pond and Little Lake F2 hybrids if crosses follow the laws of independent assortment and segregation. We calculated expected variation using the parental trait values, expected average F2 trait values, and sample sizes.

Trait	Crescent pond F2 trait variation expectations	Little Lake F2 trait variation expectations
Dentigerous arm width	0.016	0.017
Dentigerous arm base	0.034	0.0040
Lower jaw length	0.045	0.019
Dentigerous arm depth	0.0057	0.013
Palatine height	0.013	0.0048
Jaw closing in lever	0.0027	0.0049
Cranial height	0.00066	0.00000021
Orbit diameter	0.0000017	0.00063
Suspensorium length	0.00063	0.0000068
Ascending process length	0.031	0.00043
Jaw opening in lever	0.0086	0.0035
Nasal tissue protrusion	0.012	0.0034
Ectopterygoid	0.000000056	0.000058
Head depth	0.00029	0.000014
Maxillary head protrusion	0.043	0.041
Maxilla length	0.012	0.0050
Maxillary head height	0.026	0.00014
Pelvic girdle length	0.00020	0.0032

**TABLE E4:** Estimated marginal mean trait values and 95% HDI for F2 hybrids from a Bayesian model with the 18 craniofacial trait values as the response variable and population ID as the fixed effect.

Trait	Crescent pond	Little Lake
Dentigerous arm width	−8e‐04 (−0.01, 0.0082)	−0.00075 (−0.013, 0.011)
Dentigerous arm base	−0.00063 (−0.01, 0.0089)	−0.0069 (−0.021, 0.0065)
Lower jaw length	−0.0015 (−0.0092, 0.0069)	−0.0029 (−0.013, 0.0074)
Dentigerous arm depth	0.0013 (−0.0081, 0.01)	−0.002 (−0.014, 0.01)
Palatine height	0.00066 (−0.008, 0.0091)	8e‐04 (−0.011, 0.014)
Jaw closing in lever	0.0018 (−0.0085, 0.012)	0.00069 (−0.015, 0.016)
Cranial height	−0.003 (−0.013, 0.0067)	−0.0046 (−0.018, 0.0095)
Orbit diameter	−0.0028 (−0.01, 0.0046)	0.011 (−5e‐04, 0.024)
Suspensorium length	−0.0021 (−0.0098, 0.006)	0.0034 (−0.0071, 0.015)
Ascending process length	−0.0026 (−0.022, 0.016)	0.019 (−0.0075, 0.043)
Jaw opening in lever	−0.0095 (−0.028, 0.0069)	−0.011 (−0.037, 0.014)
Nasal tissue protrusion	0.0075 (−0.023, 0.036)	−0.0038 (−0.04, 0.034)
Ectopterygoid	−0.0035 (−0.019, 0.012)	0.0024 (−0.021, 0.023)
Head depth	−0.0025 (−0.009, 0.0037)	−0.0046 (−0.013, 0.0037)
Maxillary head protrusion	−0.0025 (−0.019, 0.015)	−0.0023 (−0.026, 0.021)
Maxilla length	−4.1e‐05 (−0.0083, 0.0088)	−0.00045 (−0.012, 0.012)
Maxillary head height	0.0021 (−0.028, 0.031)	0.01 (−0.033, 0.047)
Pelvic girdle length	0.00067 (−0.0093, 0.011)	−0.00048 (−0.014, 0.012)

**TABLE E5:** Correlation of loadings with PC axes one and two from a principal components analysis calculated with the variance and covariance estimates for 18 craniofacial traits for F2 hybrids from Crescent Pond and Little Lake.

Trait	PC1	PC2
Suspensorium length	0.75	−0.25
Palatine height	0.75	−0.26
Ascending process length	0.71	−0.21
Maxilla length	0.68	−0.48
Jaw closing in lever	0.53	−0.30
Maxillary head protrusion	0.35	−0.56
Cranial height	0.35	−0.76
Ectopterygoid	0.10	0.45
Orbit diameter	−0.058	−0.55
Head depth	−0.077	−0.85
Nasal tissue protrusion	−0.49	0.048
Jaw opening in lever	−0.57	0.22
Dentigerous arm width	−0.59	−0.77
Maxillary head height	−0.61	−0.44
Dentigerous arm base	−0.69	−0.46
Lower jaw length	−0.72	−0.41
Dentigerous arm depth	−0.74	−0.29
Pelvic girdle length	−0.79	0.089

**TABLE E6:** Contribution of the variation present in the P matrices of F2 hybrids from Crescent Pond and Little Lake to PC axes 1 and 2.

Crescent pond			Little Lake		
Trait	PC1	PC2	Trait	PC1	PC2
Ascending process length	1.47	2.26	Maxillary head height	23.6	12.6
Suspensorium length	0.527	3.55	Ascending process length	15.0	1.93
Cranial height	0.453	0.559	Nasal tissue protrusion	13.4	0.481
Maxillary head protrusion	0.375	0.524	Jaw opening in lever	9.58	3.23
Maxillary head height	0.308	3.47	Suspensorium length	4.15	0.100
Jaw closing in lever	0.290	1.94	Pelvic girdle length	4.09	1.60
Head depth	0.260	1.38	Jaw closing in lever	3.99	0.957
Palatine height	0.194	0.586	Palatine height	3.85	4.49
Maxilla length	0.142	0.405	Maxillary head protrusion	3.21	9.47
Dentigerous arm depth	0.0765	1.45	Dentigerous arm base	2.54	2.23
Pelvic girdle length	0.0650	2.77	Cranial height	2.52	5.11
Lower jaw length	0.0438	0.799	Maxilla length	2.17	5.44
Orbit diameter	0.0328	3.25	Dentigerous arm depth	2.03	0.871
Dentigerous arm width	0.0104	0.0856	Dentigerous arm width	1.27	6.00
Ectopterygoid	0.00823	2.98	Lower jaw length	1.24	0.352
Jaw opening in lever	0.00341	3.76	Ectopterygoid	0.140	4.35
Nasal tissue protrusion	0.00122	3.98	Head depth	0.130	1.77
Dentigerous arm base	0.00116	0.471	Orbit diameter	0.120	2.04
Total variation	7%	37%		93%	63%

## APPENDIX F INVESTIGATING BIASES IN THE SSI RADIATING P MATRIX

To explore if P matrix patterns associated with the SSI radiating group were primarily driven by the inclusion of specialist species with the generalist pupfish, we investigated if SSI sympatric generalist estimates of variation/covariation were consistently divergent from SSI sympatric specialist estimates across traits. We used chi‐squared tests to investigate if generalist variation/covariation estimates were divergent across more traits than expected by chance (i.e., count data), and we used linear models to investigate if the magnitude of difference between generalist and specialist variation/covariation estimates was consistently greater across traits than the magnitude of differences between specialists' estimates.

The chi‐squared analyses indicate that the number of traits for which generalist variance values were either most similar to or most divergent from specialist pupfish species was not different from the numbers expected by chance (χ^2^ = 0, df = 2, *p*‐value = 1; Table F1). Similarly, the number of traits for which generalist covariance estimates were most similar to or most divergent from specialist estimates, also did not significantly vary from the number expected by chance (χ^2^ = 0.77, df = 2, *p*‐value = .68; Table F2).

To investigate if the magnitude of difference between generalists and specialists was consistently different across traits, we used linear models with differences in variation for each trait or differences in covariation for each trait pair as the response variable, and species comparison as the predictor variable. Both models suggest that the magnitude of differences between variation (LM: sum of squares: 0.002, df = 2, *F* = 0.35, *p*‐value = .71) or covariation estimates (LM: sum of squares: 0.00081, df = 2, *F* = 1.85, *p*‐value = .16) do not differ across species comparisons.

**TABLE F1:** Expected and observed number of traits where (1) generalist estimates of variation were most similar to or most divergent from specialist estimates and (2) specialist variation estimates were most similar to or most divergent from one another.

	Expected		Observed	
	Most divergent	Most similar	Most divergent	Most similar
Generalist and scale‐eater	5	5	5	5
Generalist and snail‐eater	6	6	6	6
Scale‐eater and snail‐eater	7	7	7	7

**TABLE F2:** Expected and observed number of traits where (1) generalist estimates of covariation were most similar to or most divergent from specialist estimates and (2) specialist covariation estimates were most similar to or most divergent from one another.

	Expected		Observed	
	Most divergent	Most similar	Most divergent	Most similar
Generalist and scale‐eater	106	106	108	104
Generalist and snail‐eater	105	105	108	102
Scale‐eater and snail‐eater	95	95	90	100
